# Bismuth subsalicylate, probiotics, rifaximin and vaccines for the prevention of travelers’ diarrhea: a systematic review and network meta-analysis

**DOI:** 10.3389/fphar.2024.1361501

**Published:** 2024-04-18

**Authors:** Hao Fan, I-Chun Liu, Lei Gao, Lanka Wu

**Affiliations:** ^1^ School of Tourism and Service Management, Chongqing University of Education, Chongqing, China; ^2^ College of Humanities and Social Sciences, Yuan Ze University, Taoyuan, Taiwan; ^3^ School of Tourism and Hotel Management, University of Sanya, Sanya, China; ^4^ School of Culture and Tourism, Chongqing City Management College, Chongqing, China

**Keywords:** prevention modalities, systematic review, network meta-analysis, travelers’, diarrhea

## Abstract

**Background:** Bismuth subsalicylate (BSS), probiotics, rifaximin, and vaccines have been proposed as preventive modalities for patients with travelers’ diarrhea (TD), but their comparative effectiveness for prevention has rarely been studied. We aimed to perform a systematic review and network meta-analysis to test whether one of these modalities is more effective than the others in reducing the incidence of TD.

**Methods:** We searched Pubmed, Embase, the Cochrane Central Register of Controlled Trials (CENTRAL), Web of Science, and clinical registries from inception of the databases through 18 November 2023, without language restriction, for randomized controlled trials (RCTs) evaluating the efficacy of BSS, probiotics, rifaximin, and vaccines in preventing TD. The primary outcome was the incidence of TD and the safety outcome was the incidence of adverse events. The relative ratio (RR) was used to assess the effect of the modalities, and RR estimates between any two of the modalities were calculated and pooled using a frequentist network meta-analysis model.

**Results:** Thirty-one studies (recruiting 10,879 participants) were included in the analysis. Sixteen were judged to have a low risk of bias. In the aggregate analysis, BSS and rifaximin were more effective than placebo and other treatment modalities, which was further confirmed in the individual analysis. The comparison between rifaximin and placebo achieved high confidence, while the comparisons between BSS and placebo, ETEC and probiotics, and rifaximin and vaccines achieved moderate confidence. BSS had a higher rate of adverse events compared with other treatments.

**Conclusion:** Rifaximin had a relative lower TD incidence and lower adverse event rate, and the evidence was with moderate confidence.

**Systematic Review Registration:** https://osf.io/dxab6, identifier.

## Introduction

Traveler’s diarrhea is a common medical condition that affects at least 60% of people who travel (Ng et al., 2017; Riddle et al., 2017). While it is usually non-fatal and self-healing, it can cause severe symptoms such as fever, vomiting, abdominal pain or cramps, and dehydration, which can disrupt travel plans or require hospitalization (Steffen et al., 2015; Giddings et al., 2016).

Antibiotics and adequate hydration are recommended for treating TD due to strong evidence. However, evidence for the effectiveness of prevention methods is not convincing, and the comparative effectiveness between treatment modalities is unknown. This causes confusion for clinicians and individuals planning trips to high TD risk destinations.

Bismuth subsalicylate (BSS), the active ingredient in adult formulations of Pepto-Bismol, is the primary agent studied for the prevention of TD. According to reports, taking 2 chewable tablets of BSS 4 times per day reduces the incidence of TD by approximately 50% (Budisak and Abbas, 2023).

TD is caused by bacterial infection, with the most commonly reported pathogens being *Escherichia coli* (ETEC), *Campylobacter* jejuni, *Salmonella* species, and *Shigella* species (Riddle et al., 2016, 2017; Ng et al., 2017). Therefore, antibiotics and antimicrobials (probiotics and prebiotics) are proposed for the treatment of TD (Riddle et al., 2017). Two meta-analyses have confirmed the efficacy of rifaximin in preventing TD (Traveler’s Diarrhea) (Hu et al., 2012; Ng et al., 2017). Additionally, a recent meta-analysis published in 2018 concluded that probiotics are statistically significant in TD prophylaxis (Bae, 2018). Vaccines against the common pathogen ETEC are also proposed for the preventive treatment of TD. ETEC bacteria adhere to the lining of the gut and secrete either one or both types of enterotoxins: the heat-labile toxin (LT) and the heat-stable toxin. Different strains of ETEC can be further characterized based on the antigens expressed on the cell surface, such as the colonization factor (CF) (Ahmed et al., 2013). The LT and CFs are important antigens for ETEC vaccine development. The cholera vaccine contains a recombinant B subunit of the cholera toxin that is antigenically similar to the LT of ETEC. Therefore, it is also used for the prevention of TD (Walker et al., 2007).

There was limited evidence on the comparative effectiveness of these agents, particularly vaccines, probiotics, and rifaximin, in preventing TD. To address this gap, we conducted a systematic review and network meta-analysis to evaluate the efficacy of Bismuth Subsalicylate, probiotics, rifaximin, and vaccines in relation to each other for TD prevention.

## Methods

A systematic review and network meta-analysis were conducted to test the relative effectiveness of Bismuth Subsalicylate, probiotics, rifaximin, and vaccines in reducing the incidence of TD. The review was conducted in accordance with the PRISMA (Preferred Reporting Items for Systematic Reviews and Meta-Analyses) extension statement for reporting systematic reviews incorporating network meta-analyses of healthcare interventions (Hutton et al., 2015). The study used publicly available data, so no additional ethical approval is required. The systematic review was registered with the Open Science Framework (https://osf.io/dxab6).

### Literature search

Potentially eligible articles were identified from Medline, Embase, the Cochrane Central Register of Controlled Trials (CENTRAL), Web of Science, and clinical registries from the inception of the databases until 18 November 2023. Search strategies for the databases were developed (see Supplementary Table S1), and searches were performed without any language restrictions. Clinical registers, including clinicaltrials.gov and chictr.org.cn, were searched for completed studies that were not reported in peer-reviewed journals. In addition, previously published reviews were examined, and their reference lists were screened for potentially missing studies.

### Selection of studies

After conducting a literature search, two reviewers independently screened the retrieved articles. The screening process was conducted first at the title-and-abstract level and then at the full-text level. Any discrepancies in study selection were resolved through group discussion and arbitrated by a third reviewer. Only studies that met all of the following conditions were included: (1) reported as a randomized controlled trial; (2) included healthy adults over the age of 18 who planned to travel and took preventative measures; (3) assessing the effectiveness of various preventative measures, including bismuth subsalicylate, probiotics, rifaximin, and vaccines; (4) reporting the incidence of TD after travel to high-risk areas.

### Study outcomes

The study’s primary outcome was the incidence of TD. TD was defined as the passage of at least three unformed stools within a 24-hour period accompanied by at least one of the following conditions: abdominal pain or cramps, nausea, vomiting, fever (≥37.8°C), fecal urgency, passage of gross blood or mucus in stool, tenesmus, or moderate to severe increase in intestinal gas, according to our previous systematic review (Fan et al., 2022) and the American College of Gastroenterology (AGC) guideline (Riddle et al., 2016). The safety outcome will be treatment-related adverse events.

### Data extraction

Two independent reviewers performed data extraction. The eligible trials’ characteristics, including baseline parameters of participants, details of interventions and controls, and outcome measures, were extracted and presented. The authors, year of publication, total sample size of the trial, study design, and follow-up period were recorded. Baseline parameters of the participants were recorded as mean age, proportion of females, and mean scores of body mass index, and travel destination. The study recorded the dosage and frequency of treatment interventions. Missing data were obtained by contacting the authors via email. A third reviewer checked the extracted data for accuracy before preparing it for meta-analysis.

### Risk of bias assessment and grading of evidence

The Cochrane risk of bias tool (RoB 2) was used to assess the risk of bias in eligible trials (Sterne et al., 2019). The tool assessed five domains: randomization process, deviations from intended interventions, missing outcome data, measurement of the outcome, and selection of the reported result. The RoB 2 tool provided an overall evaluation of each trial, classifying it as having low risk of bias, high risk of bias, or some concerns based on the response to signaling questions for each domain.

The Confidence in Network Meta-Analysis (CINeMA) tool was used to grade the evidence. This tool is based on a methodological framework that assesses evidence of network meta-analysis in six domains: within-study bias, reporting bias, indirectness, imprecision, heterogeneity, and incoherence (Nikolakopoulou et al., 2020).

### Statistical analysis

The main objective of this study was to compare the incidence of TD when treated with bismuth subsalicylate, probiotics, rifaximin, and vaccines. To achieve this, we conducted a frequentist-approach network meta-analysis to perform pairwise comparisons since there is a lack of head-to-head comparison between these treatments. Placebo control was used as a common comparator, and the treatment effects were calculated relative to the placebo control. Pairwise comparisons were made based on the calculated estimates using relative ratios (RRs), where a lower value of RR indicated a better treatment effect. The analysis was performed using a random-effect model. The Surface Under the Cumulative Ranking curve (SUCRA) score was estimated using the network meta-analysis model. This provides information on the probability of a treatment being the best among all treatments (Daly et al., 2019).

To test the robustness of the results, two subgroup analyses were conducted. The first analysis focused on studies with low RoB, while the second analysis focused on studies with some concerns or high RoB. The results from these two subgroups were compared to determine whether RoB had an impact on the study results. We conducted separate analyses on participants traveling to Mexico and those traveling to other regions. This was done because many studies have tested the preventive effects of treatments specifically for those traveling to Mexico. We compared the results from these two subgroups to determine if travel destination had an impact on the study outcomes.

Consistency of the network meta-analysis was assessed by comparing results from direct and indirect evidence. A significant inconsistency was considered when the z-test indicated a *p*-value less than 0.05. Global heterogeneity of the network meta-analysis was examined using Cochran’s Q test and the *I* ^*2*^ statistics. An *I* ^*2*^ value less than 40% was considered unimportant heterogeneity (Higgins, 2011).

## Results

### Characteristics of the included RCTs

**FIGURE 1:**
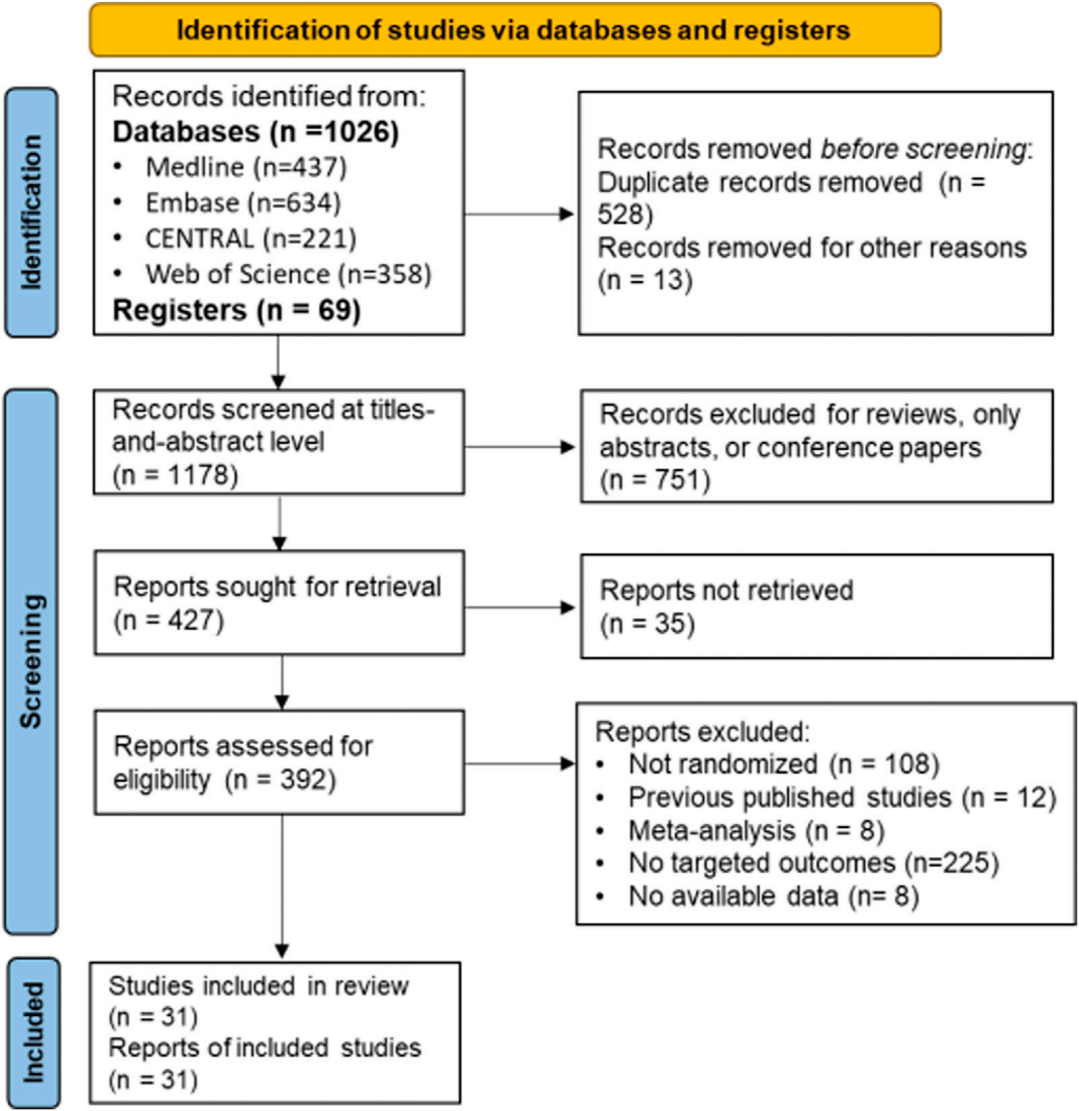
Study flowchart.

The characteristics of the included RCTs are summarized in Figure 1, which presents the process and results of the literature search and study selection. A total of 1,095 records were found during the literature search, and 31 trials (recruiting 10,879 participants) were included in the analysis (de dios Pozo-Olano et al., 1978; DuPont et al., 1980, 1987, 2005; Graham et al., 1983; Steffen et al., 1986; Black et al., 1989; Kollaritsch and Wiedermann, 1989; Oksanen et al., 1990; Peltola et al., 1991; Kollaritsch et al., 1993; Katelaris et al., 1995; Scerpella et al., 1995; Hilton et al., 1997; Wiedermann et al., 2000; Leyten et al., 2005; Briand et al., 2006; McKenzie et al., 2007, McKenzie et al., 2008; Sack et al., 2007; Armstrong et al., 2010; Drakoularakou et al., 2010; Martinez-Sandoval et al., 2010; Flores et al., 2011; Virk et al., 2013; Zanger et al., 2013; Krokowicz et al., 2014; Hasle et al., 2017; Savarino et al., 2019; Anu et al., 2023; Maier et al., 2023). Table 1 shows the characteristics of the included studies.

**TABLE 1:** Trial characteristics.

Study ID	Study population	Travel destination	Mean age (range)	Female proportion (%)	Treatment (no.)	Control (no.)	Mean treatment duration	Overall RoB
Bismuth subsalicylate								
DuPont et al. (1980)	US	Mexico	NA	NA	Bismuth subsalicylate (62)	Placebo (66)	21 days	Low risk of bias
DuPont et al. (1987)	US	Mexico	27.9	61	Bismuth subsalicylate (114)	Placebo (58)	21 days	Low risk of bias
Graham et al. (1983)	US	Provided ETEC challenge	NA	NA	Bismuth subsalicylate (15)	Placebo (16)	3 days	Some concerns
Steffen et al. (1986)	Switzerland	Varied destinations, hot climates	36.5	46	Bismuth subsalicylate (77)	Placebo (82)	12–28 days	Some concerns
Rifaximin								
Armstrong et al. (2010)	US	Turkey	36	12	Rifaximin (48)	Placebo (47)	14 days	Low risk of bias
DuPont et al. (2005)	Mexico	Mexico	NA	NA	Rifaximin (54)	Placebo (54)	14 days	Low risk of bias
Flores et al. (2011)	US	Mexico	25 (18–67)	55	Rifaximin (50)	Placebo (48)	14 days	Low risk of bias
Black et al. (1989)	Danish	Egypt	51	NA	Probiotics (47)	Placebo (47)	14 days	Some concerns
Briand et al. (2006)	French	West Africa or Asia	38 (36–40)	51.7	Probiotics (79)	Placebo (72)	14 days	Low risk of bias
de dios Pozo-Olano et al. (1978)	US	Mexico	NA	NA	Probiotics (26)	Placebo (24)	8 days	Some concerns
Drakoularakou et al. (2010)	British	Varied destinations	38.5	42.7	Probiotics (81)	Placebo (78)	>7 days	Low risk of bias
Martinez-Sandoval et al. (2010)	Mexico	Mexico	24 (18–75)	64.7	Rifaximin (99)	Placebo (102)	14 days	Low risk of bias
Zanger et al. (2013)	German	Southeast Asia	29 (24–37)	51.9	Rifaximin (122)	Placebo (117)	28 days	Low risk of bias
Probiotics								
Hasle et al. (2017)	Norwegian	Varied destinations with high risks of TD	43	51.3	Probiotics (167)	Placebo (167)	14 days	Low risk of bias
Hilton et al. (1997)	US	Varied destinations	50 (17–80)	47.7	Probiotics (126)	Placebo (119)	21 days	Some concerns
Katelaris et al. (1995)	British	Belize	NA	NA	Probiotics (181)	Placebo (101)	21 days	Some concerns
Kollaritsch and Wiedermann (1989)	Austrian	Varied destinations, hot climates	NA	NA	Probiotics (1148)	Placebo (712)	23 days	Some concerns
Kollaritsch et al. (1993)	Austrian	Varied destinations, hot climates	NA	NA	Probiotics (655)	Placebo (361)	21 days	Low risk of bias
Krokowicz et al. (2014)	Polish	Varied destinations	NA	NA	Probiotics (22)	Placebo (20)	>3 days	Low risk of bias
Oksanen et al. (1990)	Finland	Turkey	43.8 (10–80)	NA	Probiotics (373)	Placebo (383)	14 days	Some concerns
Virk et al. (2013)	US	High TD risk areas	48.7	53.1	Probiotics (94)	Placebo (102)	21 days	Low risk of bias
Vaccines								
Anu et al. (2023)	Finland	West Africa	46.4	72	Oral ETEC vaccine (374)	Placebo (375)	14 ± 7 days	Low risk of bias
Leyten et al. (2005)	Germany	Southeast Asia or West Africa	39.5	46.2	Oral cholera vaccine (69)	Placebo (65)	>14 days	Low risk of bias
Maier et al. (2023)	US	Guatemala or Mexico	NA	NA	Oral ETEC vaccine (705)	Placebo (701)	28 days	Low risk of bias
McKenzie et al. (2007)	US	NA	32	46	Transcutaneous ETEC vaccine (27)	Placebo (20)	77 days	Some concerns
McKenzie et al. (2008)	US	NA	30	38.4	Transcutaneous ETEC vaccine (27)	Placebo (20)	77 days	Some concerns
Peltola et al. (1991)	Finland	Agadir or Morocco	NA	NA	Oral cholera vaccine (307)	Placebo (308)	30 days	Some concerns
Sack et al. (2007)	US	Antigua, Guatemala or Cuernavaca, Mexico	34.6	63.5	Oral ETEC vaccine (330)	Placebo (341)	28 days	Low risk of bias
Savarino et al. (2019)	US	Provided ETEC challenge	33.4	19.4	Oral ETEC vaccine (24)	Placebo (12)	3 days	Some concerns
Scerpella et al. (1995)	US	Mexico	NA	NA	Oral cholera vaccine (251)	Placebo (251)	10 days	Some concerns
Wiedermann et al. (2000)	Finland	Morocco	NA	NA	ETEC + Cholera vaccine (121)	Placebo (66)	7–21 days	Low risk of bias

NA, not applicable; RoB, risk of bias; TD, travelers’ diarrhea.

Of these studies, sixteen were evaluated as having a low risk of bias, while fifteen were classified as having some concerns. Supplementary Figure S1 displays a detailed assessment of the risk of bias. The most common concerns were deviations from the intended interventions in eight studies and missing outcome data in eleven studies.

### Incidence of TD

**FIGURE 2:**
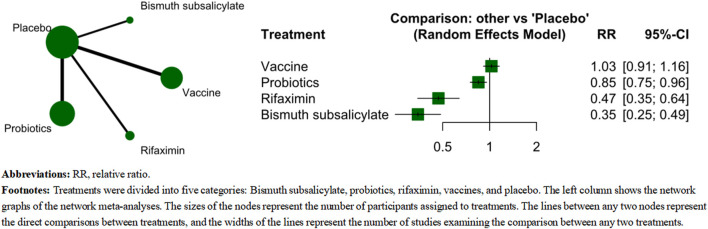
The comparative effectiveness of category-level analysis.

In this aggregate-level network meta-analysis, we included all 31 studies, comparing five categories of treatments: bismuth subsalicylate (209 participants), placebo (5,034 participants), probiotics (2,977 participants), rifaximin (373 participants), and vaccine (2,286 participants). Figure 2 displays the net-graph of this network meta-analysis. In the study, Bismuth subsalicylate, rifaximin, and probiotics were found to significantly reduce the incidence of TD when compared to the placebo (Figure 2). Bismuth subsalicylate was the most effective treatment (SUCRA score, 0.972) according to Table 2, which shows the results of pairwise comparisons. Bismuth subsalicylate was found to be more effective in reducing TD incidence than probiotics and vaccines (Table 2). There was no evidence of inconsistency between direct and indirect estimates. The test of heterogeneity showed slight heterogeneity (*I* ^*2*^ = 40.2%, tau2 = 0.015, Cochran’s Q = 45.2).

**TABLE 2:** Pairwise comparison between probiotics and rifaximin.

Bismuth subsalicylate				
0.74 (0.47–1.16)	Rifaximin			
0.41 (0.29–0.58)	0.55 (0.40–0.77)	Probiotics		
0.35 (0.25–0.49)	0.47 (0.35–0.64)	0.85 (0.75–0.96)	Placebo	
0.34 (0.24–0.48)	0.46 (0.33–0.63)	0.82 (0.70–0.98)	0.97 (0.87–1.09)	Vaccine

The top half showed the estimates of direct comparisons between two treatments, and the bottom half showed the estimates of network meta-analysis. Comparisons between treatments should be read from left to right, and the comparison estimate is in the cell between the column-defining treatment and the row-defining treatment. RRs>1 favors row-defining treatment. Green color box suggests a significant difference between the two treatments.

**FIGURE 3:**
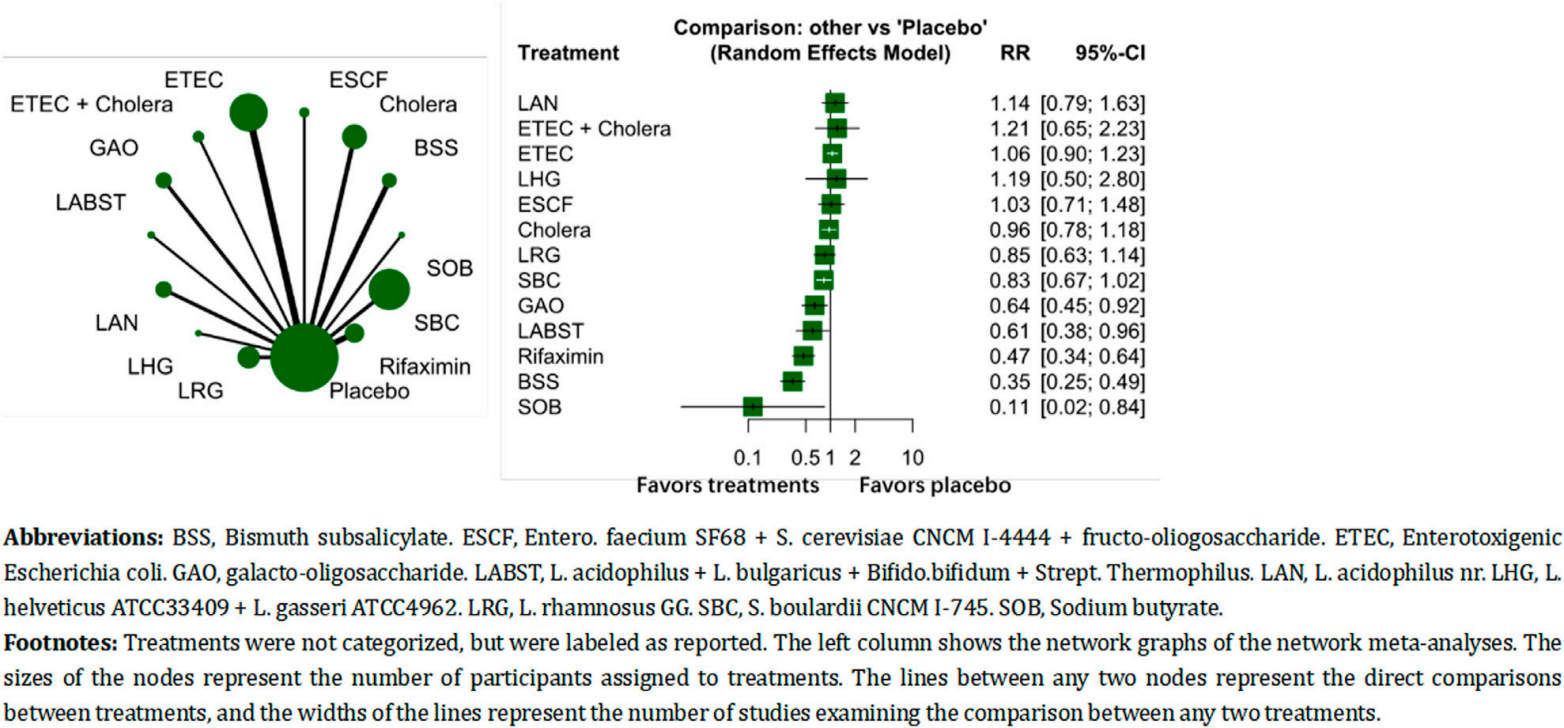
The comparative effectiveness of differential individual treatments.

The individual-level network meta-analysis included 31 studies, comparing 14 treatments: (BSS, *n* = 209), cholera vaccines (*n* = 627), *Entero. faecium* SF68 + *S. cerevisiae* CNCM I-4444 + *fructo-oliogosaccharide* (ESCF, *n* = 94), enterotoxigenic *Escherichia coli* (ETEC, *n* = 1,538), ETEC + cholera vaccines (*n* = 121), galacto-oligosaccharide (GAO, *n* = 248), L. *acidophilus* + L. *bulgaricus* + *Bifido.bifidum* + *Strept. Thermophilus* (LABST, *n* = 47), (LAN, *n* = 260), (LHG, *n* = 26), (LRG, *n* = 499), Placebo (*n* = 5,054), Rifaximin (*n* = 373), S. *boulardii* CNCM I-745 (SBC, *n* = 1803), and sodium butyrate (SOB, *n* = 22). The result showed that SOB, BSS, and rifaximin ranked the most effective treatments in the analysis (Figure 3). In pairwise comparisons, BSS was superior than most of the other treatments (Table 3). The test of heterogeneity showed unimportant heterogeneity (*I* ^*2*^ = 39.7%, tau^2^ = 0.0174, Cochran’s Q = 31.51).

**TABLE 3:** Pairwise comparisons of differential probiotics and rifaximin.

SOB													
0.33 (0.04–2.51)	BSS												
0.24 (0.03–1.84)	0.74 (0.47–1.17)	Rifaximin											
0.19 (0.02–1.47)	0.57 (0.32–1.01)	0.77 (0.44–1.35)	LABST										
0.18 (0.02–1.36)	0.54 (0.33–0.88)	0.73 (0.46–1.17)	0.94 (0.53–1.69)	GAO									
0.14 (0.02–1.03)	0.42 (0.28–0.62)	0.57 (0.39–0.82)	0.73 (0.44–1.21)	0.77 (0.51–1.17)	SBC								
0.13 (0.02–1.01)	0.41 (0.26–0.64)	0.55 (0.36–0.85)	0.71 (0.41–1.23)	0.76 (0.48–1.20)	0.98 (0.68–1.40)	LRG							
0.12 (0.02–0.89)	0.36 (0.24–0.54)	0.49 (0.34–0.71)	0.63 (0.38–1.05)	0.67 (0.44–1.01)	0.87 (0.64–1.16)	0.89 (0.62–1.27)	Cholera						
0.11 (0.02–0.84)	0.35 (0.25–0.49)	0.47 (0.34–0.64)	0.61 (0.38–0.96)	0.64 (0.45–0.92)	0.83 (0.67–1.02)	0.85 (0.63–1.14)	0.96 (0.78–1.18)	Placebo					
0.11 (0.01–0.85)	0.34 (0.21–0.56)	0.46 (0.28–0.74)	0.59 (0.33–1.06)	0.63 (0.38–1.04)	0.81 (0.53–1.23)	0.83 (0.52–1.32)	0.94 (0.62–1.42)	0.97 (0.68–1.40)	ESCF				
0.10 (0.01–0.85)	0.29 (0.12–0.73)	0.40 (0.16–0.98)	0.51 (0.19–1.35)	0.54 (0.21–1.37)	0.70 (0.29–1.69)	0.72 (0.29–1.77)	0.81 (0.33–1.95)	0.84 (0.36–1.99)	0.86 (0.34–2.19)	LHG			
0.11 (0.01–0.80)	0.33 (0.23–0.48)	0.44 (0.31–0.63)	0.57 (0.35–0.93)	0.61 (0.41–0.90)	0.79 (0.61–1.02)	0.81 (0.58–1.12)	0.91 (0.70–1.18)	0.95 (0.81–1.11)	0.97 (0.66–1.44)	1.12 (0.47–2.69)	ETEC		
0.09 (0.01–0.77)	0.29 (0.14–0.58)	0.39 (0.20–0.77)	0.50 (0.23–1.08)	0.53 (0.26–1.08)	0.69 (0.36–1.31)	0.70 (0.36–1.39)	0.79 (0.42–1.52)	0.83 (0.45–1.53)	0.85 (0.42–1.73)	0.98 (0.34–2.82)	0.87 (0.46–1.64)	ETEC + Cholera	
0.10 (0.01–0.77)	0.30 (0.19–0.50)	0.41 (0.26–0.66)	0.53 (0.30–0.96)	0.56 (0.34–0.94)	0.73 (0.48–1.11)	0.75 (0.47–1.19)	0.84 (0.56–1.28)	0.88 (0.61–1.26)	0.90 (0.54–1.50)	1.04 (0.41–2.64)	0.93 (0.63–1.37)	1.06 (0.52–2.16)	LAN

BSS, Bismuth subsalicylate; ESCF, *Entero. faecium* SF68 + *S. cerevisiae* CNCM I-4444 + fructo-oliogosaccharide; ETEC, enterotoxigenic *Escherichia coli*; GAO, galacto-oligosaccharide; LABST, L. *acidophilus* + L. *bulgaricus* + *Bifido.bifidum* + *Strept. Thermophilus*; LAN, L. *acidophilus nr*; LHG, L. *helveticus* ATCC33409 + L. *gasseri* ATCC4962; LRG, L. *rhamnosus* GG; SBC, S. *boulardii* CNCM I-745; SOB, Sodium butyrate.

The top half showed the estimates of direct comparisons between two treatments, and the bottom half showed the estimates of network meta-analysis. Comparisons between treatments should be read from left to right, and the comparison estimate is in the cell between the column-defining treatment and the row-defining treatment. RRs>1 favors row-defining treatment. Green color box suggests a significant difference between the two treatments.

### Subgroup analysis

In the category-level analysis, the first subgroup analysis revealed that BSS was the most effective treatment when studies with high risk of bias or some concerns were excluded (Supplementary Figure S2). However, when studies with high risk of bias or some concerns were included, rifaximin was found to be the most effective treatment (Supplementary Figure S3). In the individual-level analysis, BSS was ranked as the most effective treatment when studies with a high risk of bias or some concerns were excluded (see Supplementary Figure S4). On the other hand, SOB was ranked as the most effective when studies with high risk or some concerns were included (see Supplementary Figure S5).

The second subgroup analysis in the category-level analysis showed that BSS was the most effective in participants with a destination to Mexico (see Supplementary Figures S6, S7). In the individual-level analysis, SOB was the most effective (see Supplementary Figures S8, S9).

### Confidence in evidence

Supplementary Figure S11 presents the results of evidence grading for aggregate-level analysis. The comparison between rifaximin and placebo achieved high confidence, while the comparisons between BSS and placebo, ETEC and probiotics, and rifaximin and vaccines achieved moderate confidence.

Supplementary Figure S12 displays the results of evidence grading for individual-level analysis. The remaining comparisons achieved low confidence. The majority of comparisons achieved very low to low confidence.

### Adverse events

**FIGURE 4:**
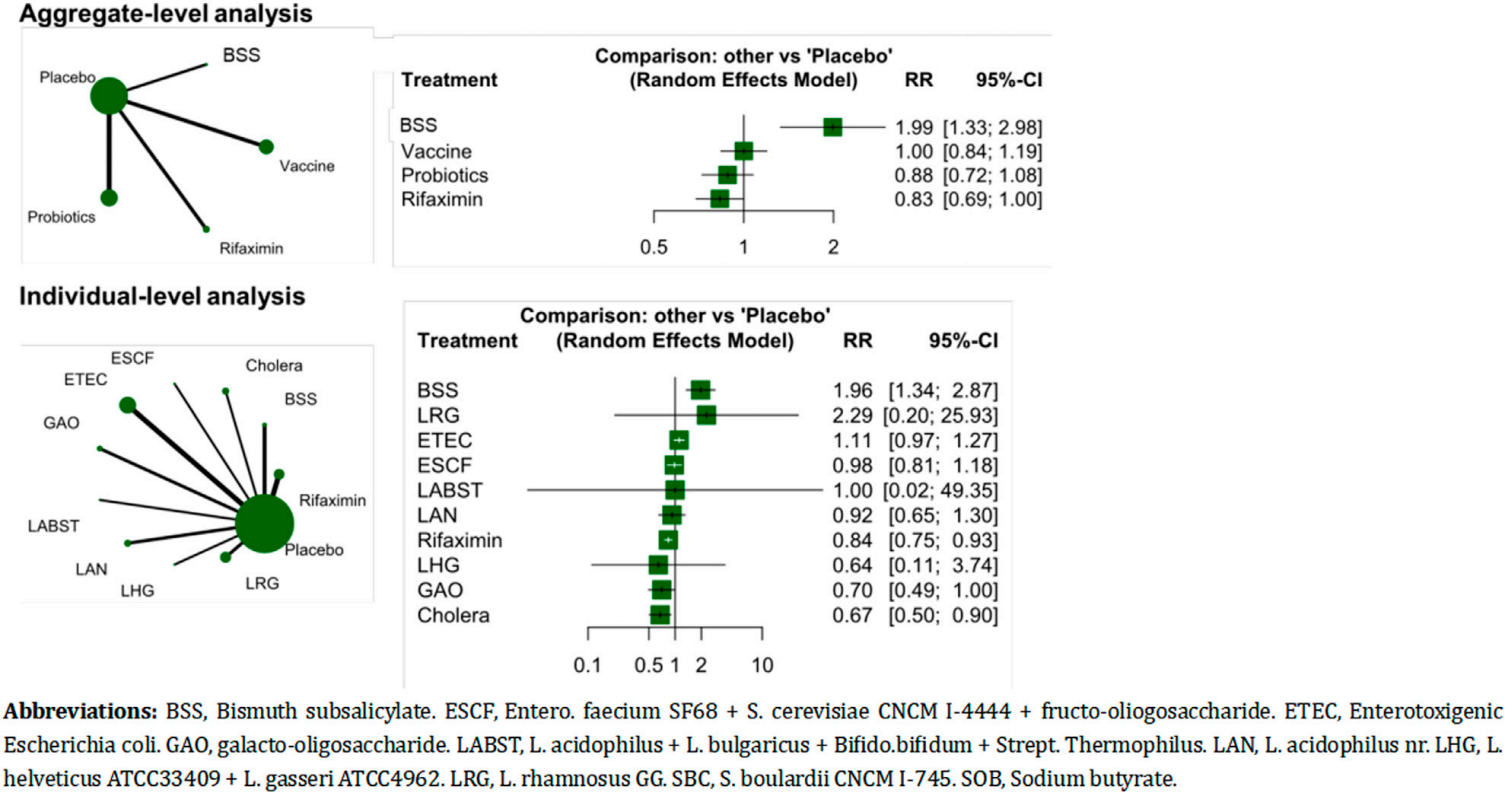
The adverse event rates of the treatments.

The analysis at the aggregate level revealed that rifaximin had a lower adverse event rate than placebo, while BSS had a significantly higher adverse event rate than placebo (*I* ^*2*^ = 20.6%, tau2 = 0.01, Cochran’s Q = 23.92) (Figure 4). At the individual level, most treatments had similar adverse event rates (*I* ^*2*^ = 0%, tau2 = 0, Cochran’s Q = 11.1) (Figure 4).

## Discussion

The network meta-analysis compared BSS, probiotics, rifaximin, and vaccines for preventing TD at both the category and individual levels. The results showed that BSS and rifaximin were both effective compared to placebo, with at least moderate certainty. Furthermore, rifaximin was more effective than vaccines and probiotics, with moderate evidence. Considering the adverse event rate, rifaximin was a better treatment option than placebo due to its lack of significant adverse events. On the other hand, BSS had a higher adverse event rate. Therefore, rifaximin had a better balance between benefit and harm.

This network meta-analysis is the first to compare the effectiveness of all currently available pharmacological treatments for preventing TD. The previous meta-analysis compared only three categories of treatments: rifaximin, probiotics, and BSS. In this network meta-analysis, we added oral ETEC vaccines and cholera vaccines. We also used the CINeMA tool to appraise the certainty of the evidence, which was not done in the previous review. The CINeMA appraisal results assist clinicians in evaluating the comparative effectiveness of a treatment and their confidence in its effectiveness.

Previous meta-analyses have suggested a marginal benefit of using antibiotics in the prevention of TD (Alajbegovic et al., 2012), in addition to our network meta-analyses. However, this is hindered in clinical practice by short follow-up periods, variability in settings and causes of acute diarrhea, and a deficiency in person-time analysis. Furthermore, there are significant differences in dosages, administration frequencies, and formulations. Additional variation can be observed in the timing and administration of these preparations in relation to various factors such as travel populations and locations, as well as concurrent antimicrobial treatment. In summary, future studies should aim to prolong the follow-up period, determine the pathogens that are suitable for rifaximin prevention of TD, study the impact of setting variability on the prevention effect, and perform person-time analysis.

We conducted an exploratory study to analyze the impact of setting variability on the preventive effect. To achieve this, we performed a subgroup analysis by separately analyzing the population with a destination to Mexico and those with a destination to other locations. This was necessary because numerous studies have been conducted in populations with a destination to Mexico. The results showed that setting variability did not affect the preventive effect of BSS and rifaximin.

Our network meta-analysis indicates a higher rate of adverse events associated with BSS in TD prevention, which limits its use in clinical practice. The adverse events commonly encountered include nausea, bitter taste, diarrhea, and dark/black stools. These events are usually mild and do not require special medical care. However, prolonged overconsumption of bismuth subsalicylate can lead to BSS toxicity, which is characterized by blackening of the tongue and teeth, fatigue, mood changes, and deterioration of mental status (Budisak and Abbas, 2023). Therefore, future studies should collect dose-effect data of adverse events and provide clinicians with the necessary information to weigh the benefits and harms.

Our study had limitations. Firstly, we may have missed eligible trials despite our comprehensive search for trials examining the effect of pharmacological treatment on TD prevention. Secondly, we were unable to study the source of infection due to insufficient background information, which may be an important factor that influences the prevention effect. Third, the allocation of participants to each treatment arm was greatly imbalanced in the network meta-analysis, which may have caused estimation bias and indicated a lack of trials in this field. Fourth, our study may have limitations in the generalizability of the results. TD is not only attributed to bacterial infection, but also to parasitic infection, especially in the tropics, or viral infection (mainly Noro virus). The treatments, antibiotic treatments or probiotics, may be appropriate for bacterial infection.

In conclusion, our network meta-analysis found that BSS and rifaximin were relatively effective treatments for the prevention of TD. Considering the grading of evidence and safety issues, rifaximin is recommended among the treatments.

## Data Availability

The original contributions presented in the study are included in the article/Supplementary Material, further inquiries can be directed to the corresponding authors.
