# Supplementary material for: Bismuth subsalicylate, probiotics, rifaximin and vaccines for the prevention of travelers’ diarrhea: a systematic review and network meta-analysis
Source: Front Pharmacol. 2024 Apr 18;15:1361501. doi: 10.3389/fphar.2024.1361501 (PMC11063717; doi:10.3389/fphar.2024.1361501)
Supplement: Supplementary file 1 [file DataSheet1.docx]

**Bismuth Subsalicylate, probiotics, rifaximin and vaccines for the prevention of travelers’ diarrhea: a systematic review and network meta-analysis**

**Outline**

[Supplementary Table 1. Search strategy 2](#_Toc153030670)

[Supplementary Figure 1. Risk of bias assessment 4](#_Toc153030671)

[Supplementary Figure 2. Subgroup analysis of studies with low risk of bias in aggregate level 5](#_Toc153030672)

[Supplementary Figure 3. Subgroup analysis of studies with some concerns in aggregate level 6](#_Toc153030673)

[Supplementary Figure 4. Subgroup analysis of studies with low risk of bias in individual treatment level 7](#_Toc153030674)

[Supplementary Figure 5. Subgroup analysis of studies with some concerns in individual treatment level 8](#_Toc153030675)

[Supplementary Figure 6. Subgroup analysis of population with destination to Mexico in aggregate treatment level 9](#_Toc153030676)

[Supplementary Figure 7. Subgroup analysis of study population with destination to other regions except Mexico in aggregate treatment level 10](#_Toc153030677)

[Supplementary 8. Subgroup analysis of study population with destination to Mexico individual treatment level 11](#_Toc153030678)

[Supplementary Figure 9. Subgroup analysis of study population with destination to other regions except Mexico in individual treatment level 12](#_Toc153030679)

# Supplementary Table 1. Search strategy

| **Pubmed** |  |
| --- | --- |
| 1. | "traveler diarrhea"[Title/Abstract] OR (("travel"[MeSH Terms] OR "travel"[All Fields] OR "traveling"[All Fields] OR "travelling"[All Fields] OR "travels"[All Fields] OR "traveled"[All Fields] OR "traveler"[All Fields] OR "traveler s"[All Fields] OR "travelers"[All Fields] OR "travelled"[All Fields] OR "traveller"[All Fields] OR "traveller s"[All Fields] OR "travellers"[All Fields]) AND "diarrhoea"[Title/Abstract]) OR "escherichia coli"[Title/Abstract] OR "diarrhea"[MeSH Terms] |
| 2. | ("randomized controlled trials as topic"[MeSH Terms] OR "randomized controlled trials as topic"[MeSH Terms] OR "randomized controlled trials as topic"[MeSH Terms] ) OR (randomized controlled trial[Title/Abstract]) |
| 3. | "meta analysis as topic"[MeSH Terms] OR "systematic review"[Title/Abstract] OR "meta-analysis"[Title/Abstract] |
| 4. | #2 OR #3 |
| 5. | "bismuth subsalicylate"[Title/Abstract] OR "probiotic*"[Title/Abstract] OR "rifaximin"[Title/Abstract] OR "vaccine"[Title/Abstract] OR "escherichia coli"[Title/Abstract] OR "cholera"[Title/Abstract] OR "bifidobacte*"[Title/Abstract] |
| 6. | #1 AND #4 AND #5 |
| **EMBASE** |  |
| 1. | ‘randomized Controlled Trial’/exp |
| 2. | ‘randomized Controlled Trials as Topic’/exp |
| 3. | ‘randomized controlled trial’:ab,ti |
| 4. | ‘controlled clinical trial’/exp |
| 5. | ‘controlled clinical trial’:ab,ti |
| 6. | (#1 OR #2 OR #3 OR #4 OR #5) AND [humans]/lim |
| 7. | ‘travel$’:ab,ti |
| 8. | ‘diarrhe$’:ab,ti |
| 9. | ‘escherichia coli’:ab,ti |
| 10. | #7 OR #8 OR #9 |
| 11. | ‘bismuth subsalicylate’:ab,ti |
| 13. | ‘probiotic*’:ab,ti |
| 14. | ‘rifaximin’:ab,ti |
| 15. | ‘vaccine’:ab,ti |
| 16. | ‘ETEC’:ab,ti |
| 17. | ‘cholera’:ab,ti |
| 18. | ‘bifidobacte*’:ab,ti |
| 19. | #11 OR #12 OR #13 OR #14 OR #15 OR #16 OR #17 OR #18 |
| 20. | #6 and #10 AND #19 |
| **Cochrane CENTRAL via OVID** |  |
| 1. | randomized controlled trial.pt. |
| 2. | controlled clinical trial.pt. |
| 3. | randomized.ab. |
| 4. | randomised.ab. |
| 5. | randomly.ab. |
| 6. | or/1-5 |
| 7. | travel$.ti,ab. |
| 8. | diarrhe$.ti,ab |
| 9. | escherichia coli.ti,ab |
| 10. | ETEC.ti,ab |
| 11. | or/7-10 |
| 12. | Bismuth subsalicylate. ti,ab |
| 13. | Probiotic*.ti,ab |
| 14. | Rifaximin.ti,ab |
| 15. | Vaccine.ti,ab |
| 16. | ETEC.ti,ab |
| 17. | Cholera.ti,ab |
| 18. | Bifidobacte*.ti,ab |
| 19. | or/12-18 |
| 20. | 6 AND 11 AND 19 |
| **Web of Science** |  |
| 1. | TS=(travel$ or diarrhea or escherichia coli or ETEC) or TI=(travel$ or diarrhea or escherichia coli or ETEC) or AB=(travel$ or diarrhea or escherichia coli or ETEC) |
| 2. | TS=(randomized controlled trial or placebo or double blind) or TI=(randomized controlled trial or placebo or double blind) or AB=(randomized controlled trial or placebo or double blind) |
| 3. | TS=(bismuth subsalicylate or probiotic* or rifaximin or vaccine or escherichia coli or cholera) or TI=( bismuth subsalicylate or probiotic* or rifaximin or vaccine or escherichia coli or cholera) or AB=( bismuth subsalicylate or probiotic* or rifaximin or vaccine or escherichia coli or cholera) |
| 4. | #1 AND #2 AND #3 |

# Supplementary Table 2. Evidence grading in aggregate treatment level network meta-analysis

| **Comparison** | **Number of studies** | **Within-study bias** | **Reporting bias** | **Indirectness** | **Imprecision** | **Heterogeneity** | **Incoherence** | **Confidence rating** |
| --- | --- | --- | --- | --- | --- | --- | --- | --- |
| Bismuth subsalicylate:Placebo | 4 | No concerns | Low risk | No concerns | No concerns | No concerns | Some concerns | Moderate |
| Placebo:Probiotics | 11 | Some concerns | Low risk | No concerns | No concerns | Major concerns | Some concerns | Low |
| Placebo:Rifaximin | 5 | No concerns | Low risk | No concerns | No concerns | No concerns | No concerns | High |
| Placebo:Vaccine | 11 | Some concerns | Low risk | No concerns | Major concerns | No concerns | Some concerns | Low |
| Bismuth subsalicylate:Probiotics | 0 | No concerns | Low risk | No concerns | No concerns | No concerns | Major concerns | Low |
| Bismuth subsalicylate:Rifaximin | 0 | No concerns | Low risk | No concerns | Major concerns | No concerns | Major concerns | Low |
| Bismuth subsalicylate:Vaccine | 0 | No concerns | Low risk | No concerns | No concerns | No concerns | Major concerns | Low |
| Probiotics:Rifaximin | 0 | No concerns | Low risk | No concerns | No concerns | No concerns | Some concerns | Moderate |
| Probiotics:Vaccine | 0 | Some concerns | Low risk | No concerns | No concerns | Major concerns | Some concerns | Low |
| Rifaximin:Vaccine | 0 | No concerns | Low risk | No concerns | No concerns | No concerns | Some concerns | Moderate |

# Supplementary Table 3. Evidence grading of individual treatment level network meta-analysis

| **Comparison** | **Number of studies** | **Within-study bias** | **Reporting bias** | **Indirectness** | **Imprecision** | **Heterogeneity** | **Incoherence** | **Confidence rating** |
| --- | --- | --- | --- | --- | --- | --- | --- | --- |
| BSS:Placebo | 4 | No concerns | Low risk | No concerns | No concerns | No concerns | Some concerns | Moderate |
| Cholera:Placebo | 3 | Some concerns | Low risk | No concerns | No concerns | No concerns | Major concerns | Low |
| ESCF:Placebo | 1 | No concerns | Low risk | No concerns | No concerns | No concerns | Major concerns | Low |
| ETEC:Placebo | 7 | No concerns | Low risk | No concerns | No concerns | No concerns | Some concerns | Moderate |
| ETEC + Cholera:Placebo | 1 | No concerns | Low risk | No concerns | Some concerns | No concerns | Major concerns | Low |
| GAO:Placebo | 2 | No concerns | Low risk | No concerns | No concerns | Some concerns | Major concerns | Low |
| LABST:Placebo | 1 | Some concerns | Low risk | No concerns | No concerns | Some concerns | Major concerns | Low |
| LAN:Placebo | 2 | Some concerns | Low risk | No concerns | No concerns | No concerns | Major concerns | Low |
| LHG:Placebo | 1 | Some concerns | Low risk | No concerns | Some concerns | Some concerns | Major concerns | Low |
| LRG:Placebo | 2 | Some concerns | Low risk | No concerns | No concerns | No concerns | Major concerns | Low |
| Placebo:Rifaximin | 5 | No concerns | Low risk | No concerns | No concerns | No concerns | No concerns | High |
| Placebo:SBC | 2 | Some concerns | Low risk | No concerns | No concerns | No concerns | Major concerns | Low |
| Placebo:SOB | 1 | Some concerns | Low risk | No concerns | No concerns | No concerns | Major concerns | Low |
| BSS:Cholera | 0 | Some concerns | Low risk | No concerns | No concerns | No concerns | Major concerns | Low |
| BSS:ESCF | 0 | No concerns | Low risk | No concerns | No concerns | No concerns | Major concerns | Low |
| BSS:ETEC | 0 | No concerns | Low risk | No concerns | No concerns | No concerns | Major concerns | Low |
| BSS:ETEC + Cholera | 0 | No concerns | Low risk | No concerns | No concerns | No concerns | Major concerns | Low |
| BSS:GAO | 0 | No concerns | Low risk | No concerns | No concerns | No concerns | Major concerns | Low |
| BSS:LABST | 0 | Some concerns | Low risk | No concerns | Some concerns | No concerns | Major concerns | Very low |
| BSS:LAN | 0 | No concerns | Low risk | No concerns | No concerns | No concerns | Major concerns | Low |
| BSS:LHG | 0 | Some concerns | Low risk | No concerns | No concerns | No concerns | Major concerns | Low |
| BSS:LRG | 0 | Some concerns | Low risk | No concerns | No concerns | No concerns | Major concerns | Low |
| BSS:Rifaximin | 0 | No concerns | Low risk | No concerns | Some concerns | No concerns | Some concerns | Low |
| BSS:SBC | 0 | Some concerns | Low risk | No concerns | No concerns | No concerns | Major concerns | Low |
| BSS:SOB | 0 | Some concerns | Low risk | No concerns | Major concerns | No concerns | Major concerns | Low |
| Cholera:ESCF | 0 | No concerns | Low risk | No concerns | No concerns | No concerns | Major concerns | Low |
| Cholera:ETEC | 0 | Some concerns | Low risk | No concerns | No concerns | No concerns | Major concerns | Low |
| Cholera:ETEC + Cholera | 0 | No concerns | Low risk | No concerns | Some concerns | No concerns | Major concerns | Low |
| Cholera:GAO | 0 | No concerns | Low risk | No concerns | Some concerns | No concerns | Major concerns | Low |
| Cholera:LABST | 0 | Some concerns | Low risk | No concerns | Some concerns | No concerns | Major concerns | Low |
| Cholera:LAN | 0 | Some concerns | Low risk | No concerns | No concerns | Some concerns | Major concerns | Low |
| Cholera:LHG | 0 | Some concerns | Low risk | No concerns | Some concerns | Some concerns | Major concerns | Very low |
| Cholera:LRG | 0 | Some concerns | Low risk | No concerns | No concerns | No concerns | Major concerns | Low |
| Cholera:Rifaximin | 0 | No concerns | Low risk | No concerns | No concerns | No concerns | Some concerns | Moderate |
| Cholera:SBC | 0 | Some concerns | Low risk | No concerns | No concerns | No concerns | Major concerns | Low |
| Cholera:SOB | 0 | Some concerns | Low risk | No concerns | No concerns | Some concerns | Major concerns | Very low |
| ESCF:ETEC | 0 | No concerns | Low risk | No concerns | No concerns | No concerns | Major concerns | Low |
| ESCF:ETEC + Cholera | 0 | No concerns | Low risk | No concerns | Some concerns | No concerns | Major concerns | Low |
| ESCF:GAO | 0 | No concerns | Low risk | No concerns | Some concerns | No concerns | Major concerns | Low |
| ESCF:LABST | 0 | Some concerns | Low risk | No concerns | Some concerns | No concerns | Major concerns | Very low |
| ESCF:LAN | 0 | No concerns | Low risk | No concerns | No concerns | Some concerns | Major concerns | Low |
| ESCF:LHG | 0 | Some concerns | Low risk | No concerns | Major concerns | No concerns | Major concerns | Very low |
| ESCF:LRG | 0 | Some concerns | Low risk | No concerns | No concerns | Some concerns | Major concerns | Low |
| ESCF:Rifaximin | 0 | No concerns | Low risk | No concerns | No concerns | No concerns | Some concerns | Low |
| ESCF:SBC | 0 | No concerns | Low risk | No concerns | No concerns | Some concerns | Major concerns | Low |
| ESCF:SOB | 0 | Some concerns | Low risk | No concerns | No concerns | Some concerns | Major concerns | Very low |
| ETEC:ETEC + Cholera | 0 | No concerns | Low risk | No concerns | Some concerns | No concerns | Major concerns | Low |
| ETEC:GAO | 0 | No concerns | Low risk | No concerns | No concerns | Some concerns | Major concerns | Low |
| ETEC:LABST | 0 | Some concerns | Low risk | No concerns | No concerns | Some concerns | Major concerns | Very low |
| ETEC:LAN | 0 | No concerns | Low risk | No concerns | No concerns | No concerns | Major concerns | Low |
| ETEC:LHG | 0 | Some concerns | Low risk | No concerns | Major concerns | No concerns | Major concerns | Low |
| ETEC:LRG | 0 | Some concerns | Low risk | No concerns | No concerns | No concerns | Major concerns | Low |
| ETEC:Rifaximin | 0 | No concerns | Low risk | No concerns | No concerns | No concerns | Some concerns | Moderate |
| ETEC:SBC | 0 | No concerns | Low risk | No concerns | No concerns | No concerns | Major concerns | Low |
| ETEC:SOB | 0 | Some concerns | Low risk | No concerns | No concerns | No concerns | Major concerns | Low |
| ETEC + Cholera:GAO | 0 | No concerns | Low risk | No concerns | Some concerns | No concerns | Major concerns | Low |
| ETEC + Cholera:LABST | 0 | Some concerns | Low risk | No concerns | Some concerns | No concerns | Major concerns | Low |
| ETEC + Cholera:LAN | 0 | No concerns | Low risk | No concerns | Some concerns | Some concerns | Major concerns | Very low |
| ETEC + Cholera:LHG | 0 | Some concerns | Low risk | No concerns | Major concerns | No concerns | Major concerns | Low |
| ETEC + Cholera:LRG | 0 | Some concerns | Low risk | No concerns | Some concerns | No concerns | Major concerns | Very low |
| ETEC + Cholera:Rifaximin | 0 | No concerns | Low risk | No concerns | No concerns | No concerns | Major concerns | Low |
| ETEC + Cholera:SBC | 0 | No concerns | Low risk | No concerns | Some concerns | No concerns | Major concerns | Low |
| ETEC + Cholera:SOB | 0 | Some concerns | Low risk | No concerns | No concerns | No concerns | Major concerns | Low |
| GAO:LABST | 0 | Some concerns | Low risk | No concerns | No concerns | Some concerns | Major concerns | Low |
| GAO:LAN | 0 | No concerns | Low risk | No concerns | No concerns | Some concerns | Major concerns | Low |
| GAO:LHG | 0 | Some concerns | Low risk | No concerns | Some concerns | No concerns | Major concerns | Very low |
| GAO:LRG | 0 | Some concerns | Low risk | No concerns | Some concerns | No concerns | Major concerns | Very low |
| GAO:Rifaximin | 0 | No concerns | Low risk | No concerns | Some concerns | No concerns | Major concerns | Low |
| GAO:SBC | 0 | No concerns | Low risk | No concerns | No concerns | Some concerns | Major concerns | High |
| GAO:SOB | 0 | Some concerns | Low risk | No concerns | Some concerns | No concerns | Major concerns | Very low |
| LABST:LAN | 0 | Some concerns | Low risk | No concerns | No concerns | Some concerns | Major concerns | Very low |
| LABST:LHG | 0 | Some concerns | Low risk | No concerns | Some concerns | No concerns | Major concerns | Very low |
| LABST:LRG | 0 | Some concerns | Low risk | No concerns | Some concerns | No concerns | Major concerns | Very low |
| LABST:Rifaximin | 0 | Some concerns | Low risk | No concerns | Some concerns | No concerns | Some concerns | Low |
| LABST:SBC | 0 | Some concerns | Low risk | No concerns | Some concerns | No concerns | Major concerns | Very low |
| LABST:SOB | 0 | Some concerns | Low risk | No concerns | Some concerns | No concerns | Major concerns | Very low |
| LAN:LHG | 0 | Some concerns | Low risk | No concerns | Major concerns | No concerns | Major concerns | Very low |
| LAN:LRG | 0 | Some concerns | Low risk | No concerns | Some concerns | No concerns | Major concerns | Very low |
| LAN:Rifaximin | 0 | No concerns | Low risk | No concerns | No concerns | No concerns | Some concerns | Moderate |
| LAN:SBC | 0 | Some concerns | Low risk | No concerns | Some concerns | No concerns | Major concerns | Very low |
| LAN:SOB | 0 | Some concerns | Low risk | No concerns | No concerns | No concerns | Major concerns | Low |
| LHG:LRG | 0 | Some concerns | Low risk | No concerns | Some concerns | No concerns | Major concerns | Very low |
| LHG:Rifaximin | 0 | Some concerns | Low risk | No concerns | No concerns | Some concerns | Some concerns | Low |
| LHG:SBC | 0 | Some concerns | Low risk | No concerns | Some concerns | No concerns | Major concerns | Low |
| LHG:SOB | 0 | Some concerns | Low risk | No concerns | No concerns | Some concerns | Major concerns | Low |
| LRG:Rifaximin | 0 | Some concerns | Low risk | No concerns | No concerns | No concerns | Major concerns | Low |
| LRG:SBC | 0 | Some concerns | Low risk | No concerns | No concerns | No concerns | Major concerns | Low |
| LRG:SOB | 0 | Some concerns | Low risk | No concerns | Some concerns | No concerns | Major concerns | Very low |
| Rifaximin:SBC | 0 | No concerns | Low risk | No concerns | No concerns | No concerns | Some concerns | Moderate |
| Rifaximin:SOB | 0 | Some concerns | Low risk | No concerns | Some concerns | Some concerns | Some concerns | Low |
| SBC:SOB | 0 | Some concerns | Low risk | No concerns | Some concerns | No concerns | Major concerns | Very low |

# Supplementary Figure 1. Risk of bias assessment


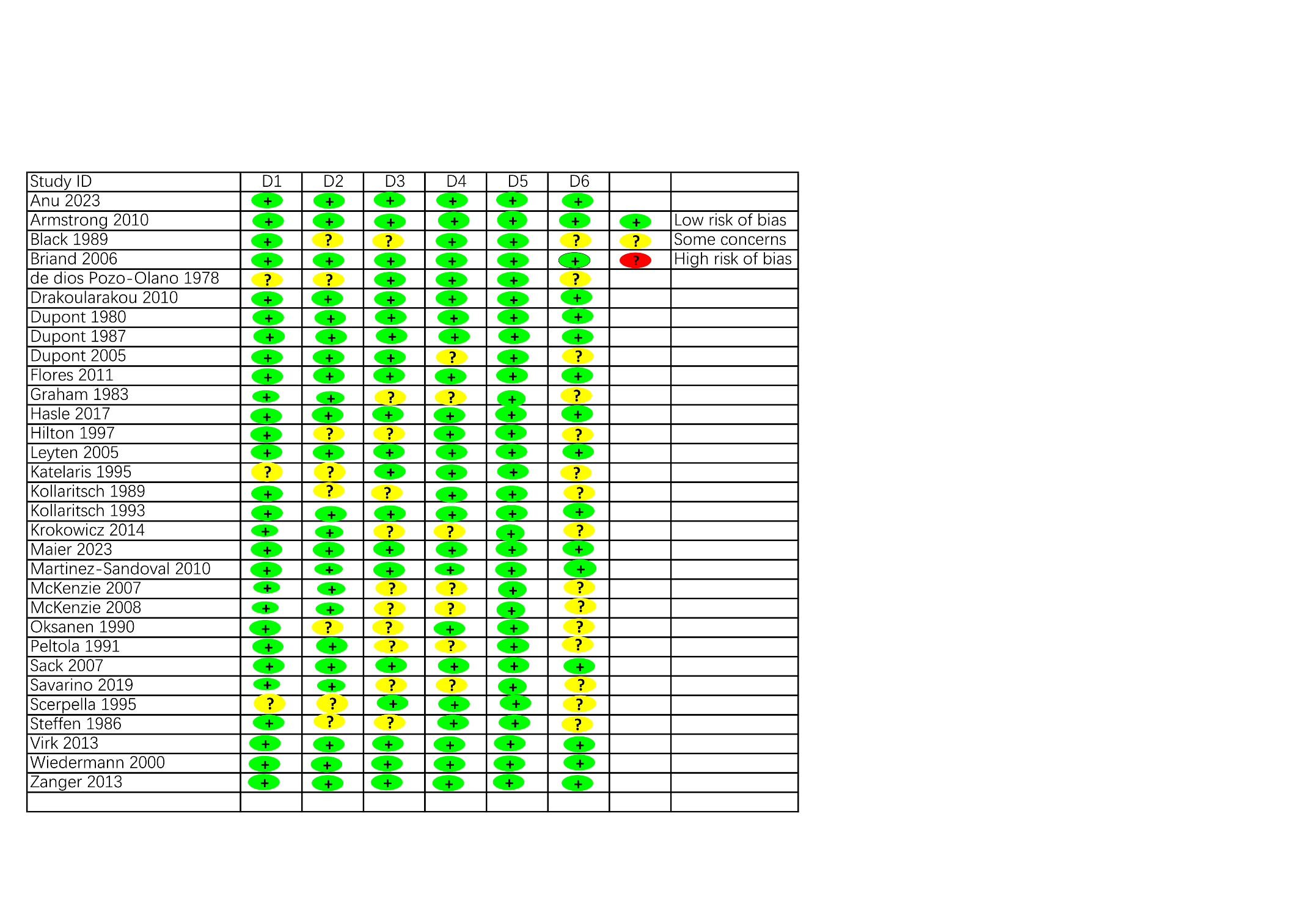


**Footnotes:** D1, Randomization process. D2, Deviations from intended interventions. D3, Missing outcome data. D4, Measurement of the outcome. D5, Selection of the reported result. D6 overall.

# Supplementary Figure 2. Subgroup analysis of studies with low risk of bias in aggregate level


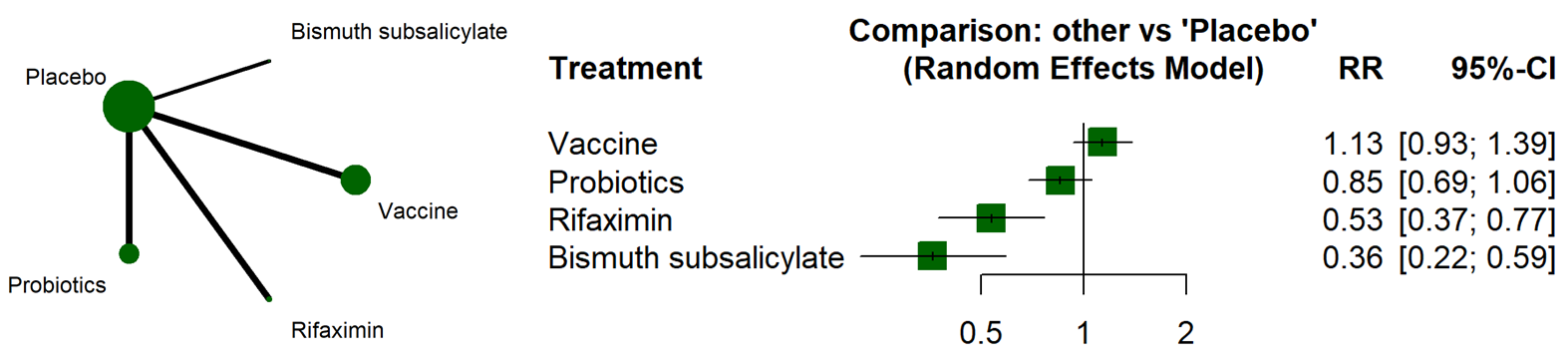


**Abbreviations:** RR, relative ratio.

**Footnotes:** We performed the subgroup analysis by including trials with low risk of bias only.

# Supplementary Figure 3. Subgroup analysis of studies with some concerns in aggregate level


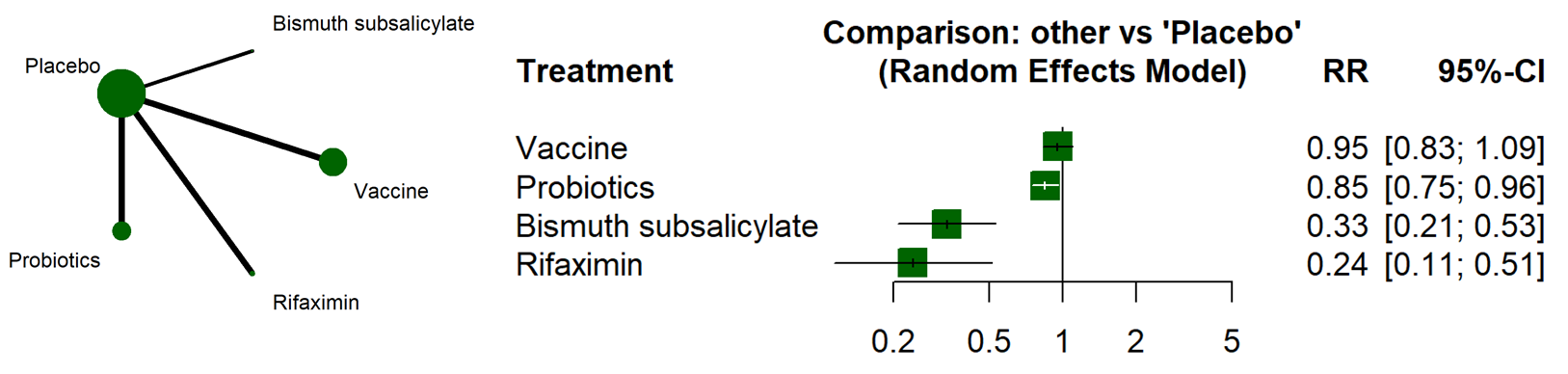


**Abbreviations:** RR, relative ratio.

**Footnotes:** We performed the subgroup analysis by including trials with low risk of bias only.

# Supplementary Figure 4. Subgroup analysis of studies with low risk of bias in individual treatment level


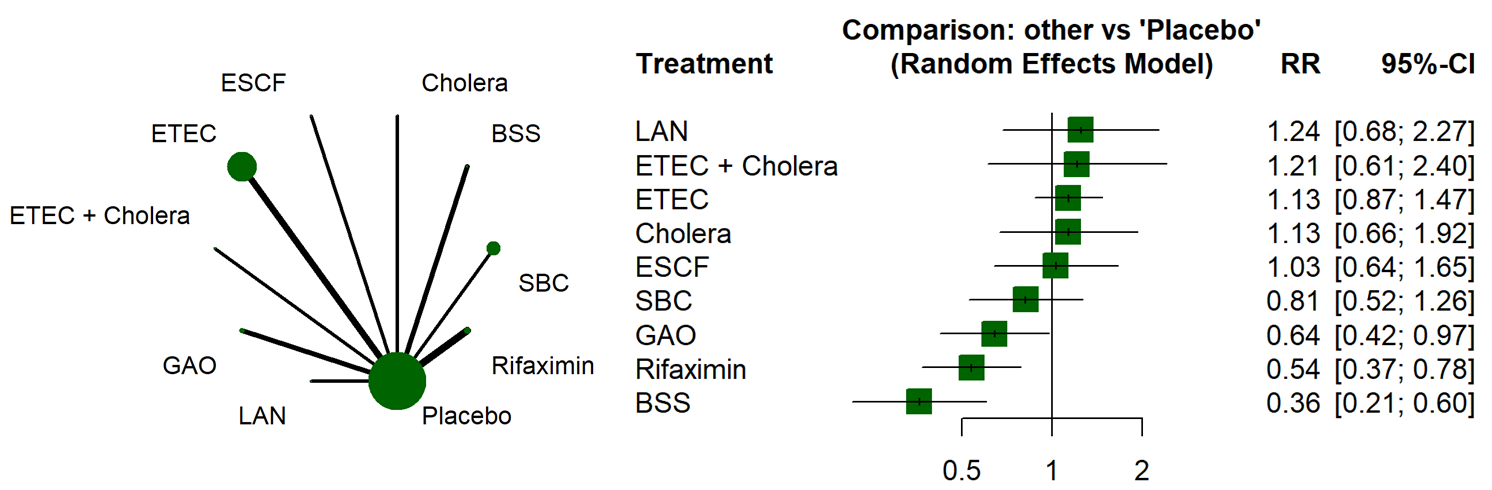


**Abbreviations:** BSS, Bismuth subsalicylate. ESCF, Entero. faecium SF68 + S. cerevisiae CNCM I-4444 + fructo-oliogosaccharide. ETEC, Enterotoxigenic Escherichia coli. GAO, galacto-oligosaccharide. LABST, L. acidophilus + L. bulgaricus + Biﬁdo.biﬁdum + Strept. Thermophilus. LAN, L. acidophilus nr. LHG, L. helveticus ATCC33409 + L. gasseri ATCC4962. LRG, L. rhamnosus GG. SBC, S. boulardii CNCM I-745. SOB, Sodium butyrate.

**Footnotes:** We performed the sensitivity analysis by including trials with low risk of bias only.

# Supplementary Figure 5. Subgroup analysis of studies with some concerns in individual treatment level


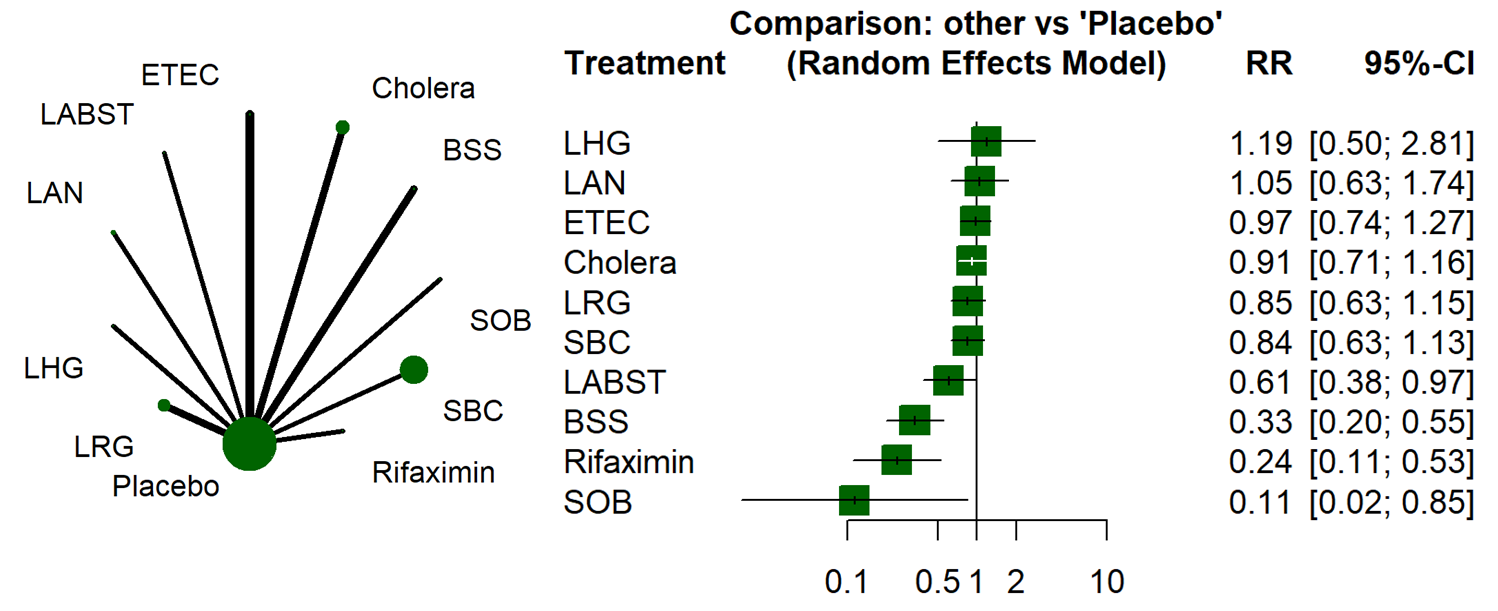


**Abbreviations:** BSS, Bismuth subsalicylate. ESCF, Entero. faecium SF68 + S. cerevisiae CNCM I-4444 + fructo-oliogosaccharide. ETEC, Enterotoxigenic Escherichia coli. GAO, galacto-oligosaccharide. LABST, L. acidophilus + L. bulgaricus + Biﬁdo.biﬁdum + Strept. Thermophilus. LAN, L. acidophilus nr. LHG, L. helveticus ATCC33409 + L. gasseri ATCC4962. LRG, L. rhamnosus GG. SBC, S. boulardii CNCM I-745. SOB, Sodium butyrate.

**Footnotes:** We performed the sensitivity analysis by including trials with some concerns only.

# Supplementary Figure 6. Subgroup analysis of population with destination to Mexico in aggregate treatment level


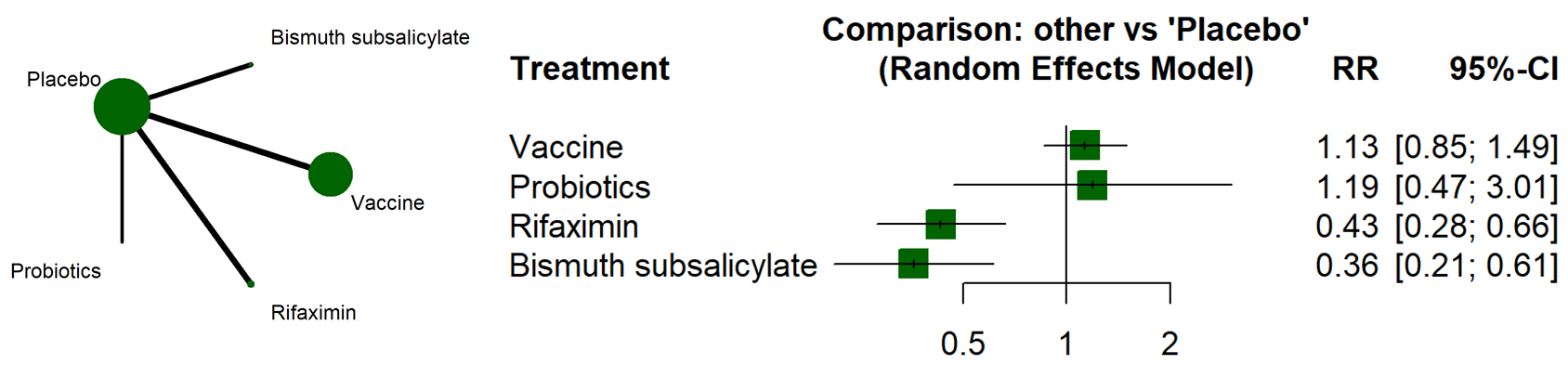


**Abbreviations:** RR, relative ratio.

**Footnotes:** We performed the subgroup analysis by including trials that reported the travel destination to Mexico.

# Supplementary Figure 7. Subgroup analysis of study population with destination to other regions except Mexico in aggregate treatment level


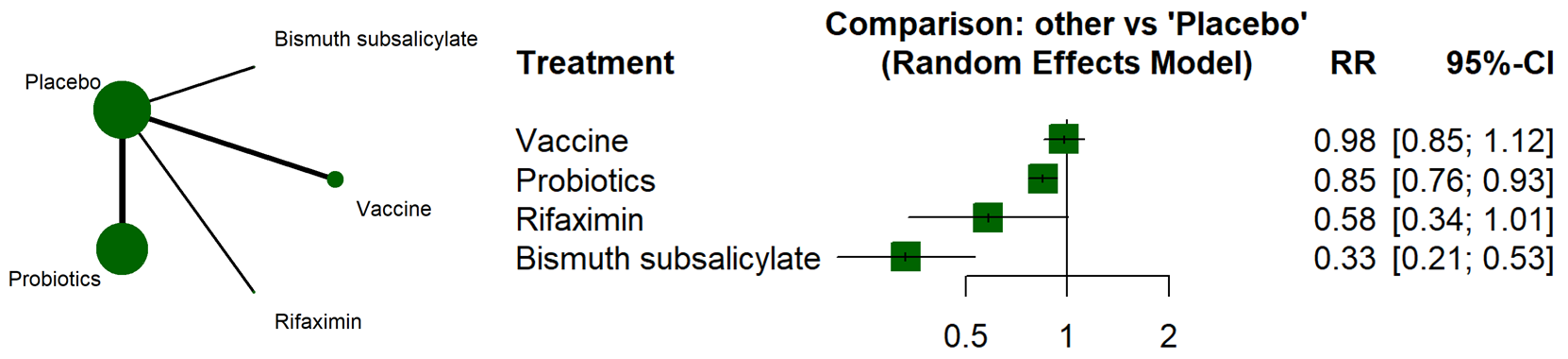


**Abbreviations:** BSS, Bismuth subsalicylate. ESCF, Entero. faecium SF68 + S. cerevisiae CNCM I-4444 + fructo-oliogosaccharide. ETEC, Enterotoxigenic Escherichia coli. GAO, galacto-oligosaccharide. LABST, L. acidophilus + L. bulgaricus + Biﬁdo.biﬁdum + Strept. Thermophilus. LAN, L. acidophilus nr. LHG, L. helveticus ATCC33409 + L. gasseri ATCC4962. LRG, L. rhamnosus GG. SBC, S. boulardii CNCM I-745. SOB, Sodium butyrate.

**Footnotes:** We performed the sensitivity analysis by including study population with destination to other regions except Mexico in aggregate treatment level.

# Supplementary 8. Subgroup analysis of study population with destination to Mexico individual treatment level


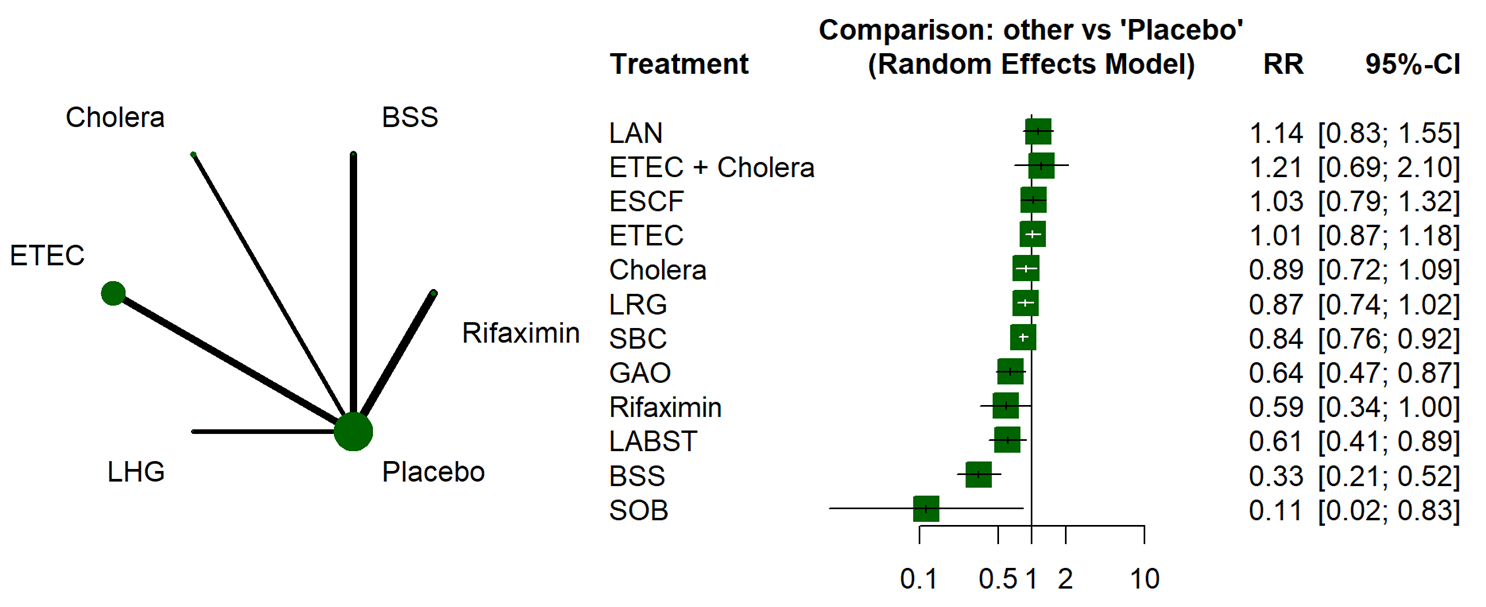


**Abbreviations:** BSS, Bismuth subsalicylate. ESCF, Entero. faecium SF68 + S. cerevisiae CNCM I-4444 + fructo-oliogosaccharide. ETEC, Enterotoxigenic Escherichia coli. GAO, galacto-oligosaccharide. LABST, L. acidophilus + L. bulgaricus + Biﬁdo.biﬁdum + Strept. Thermophilus. LAN, L. acidophilus nr. LHG, L. helveticus ATCC33409 + L. gasseri ATCC4962. LRG, L. rhamnosus GG. SBC, S. boulardii CNCM I-745. SOB, Sodium butyrate.

**Footnotes:** We performed the sensitivity analysis by including study population with destination to Mexico in individual treatment level.

# Supplementary Figure 9. Subgroup analysis of study population with destination to other regions except Mexico in individual treatment level


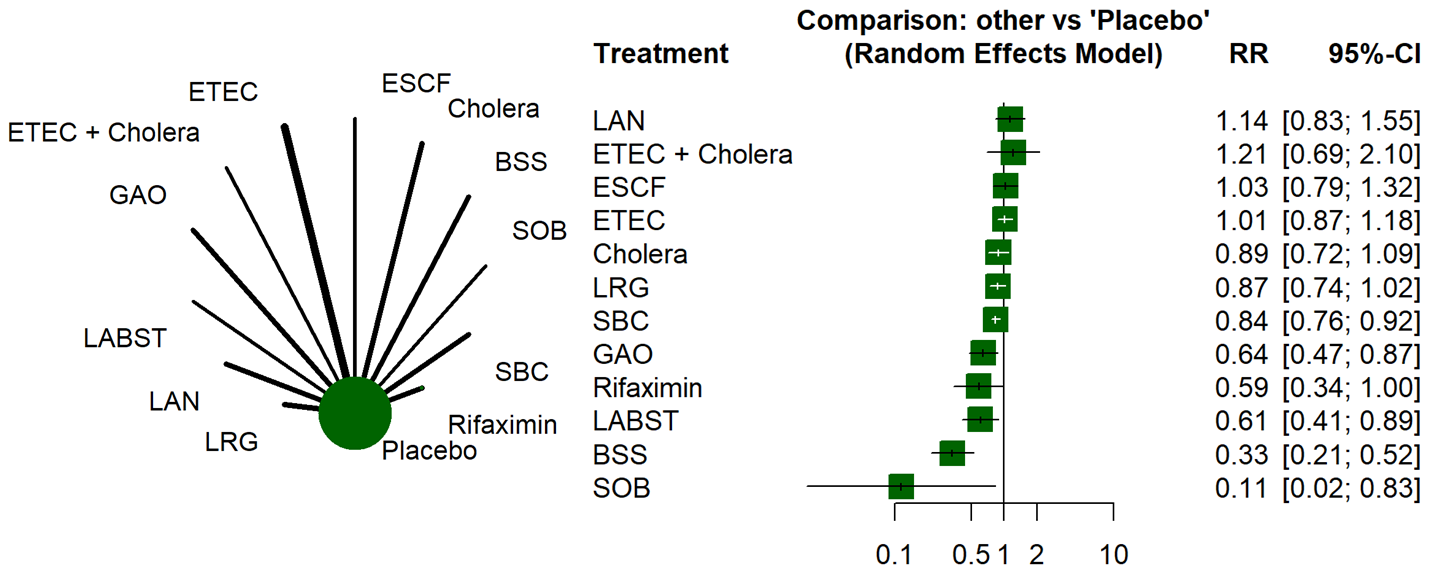


**Abbreviations:** BSS, Bismuth subsalicylate. ESCF, Entero. faecium SF68 + S. cerevisiae CNCM I-4444 + fructo-oliogosaccharide. ETEC, Enterotoxigenic Escherichia coli. GAO, galacto-oligosaccharide. LABST, L. acidophilus + L. bulgaricus + Biﬁdo.biﬁdum + Strept. Thermophilus. LAN, L. acidophilus nr. LHG, L. helveticus ATCC33409 + L. gasseri ATCC4962. LRG, L. rhamnosus GG. SBC, S. boulardii CNCM I-745. SOB, Sodium butyrate.

**Footnotes:** We performed the sensitivity analysis by including study population with destination to other regions except Mexico in individual treatment level.

# Supplementary Figure 10. Grading of evidence for aggregate-level analysis


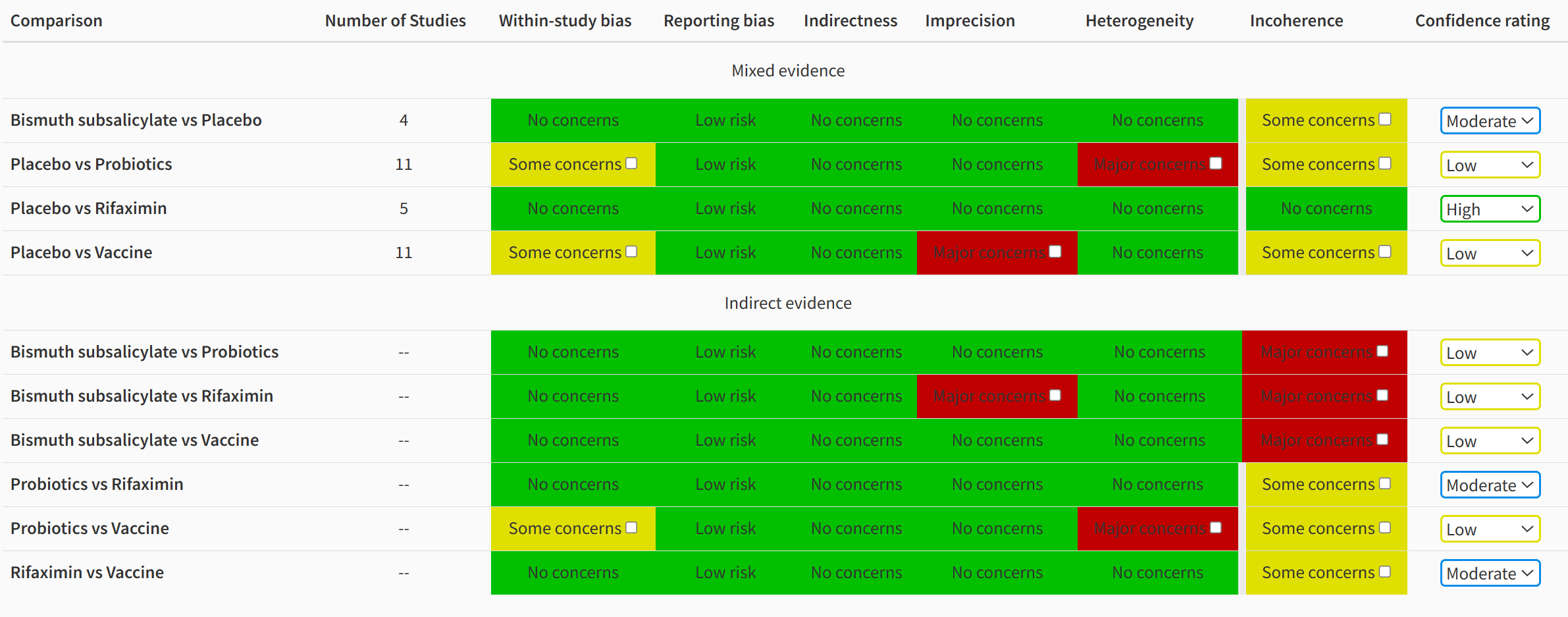


**Footnotes:** Grading of evidence was performed using Confidence in Network Meta-Analysis (CINeMA) tool.

# Supplementary Figure 11. Grading of evidence for aggregate-level analysis

**
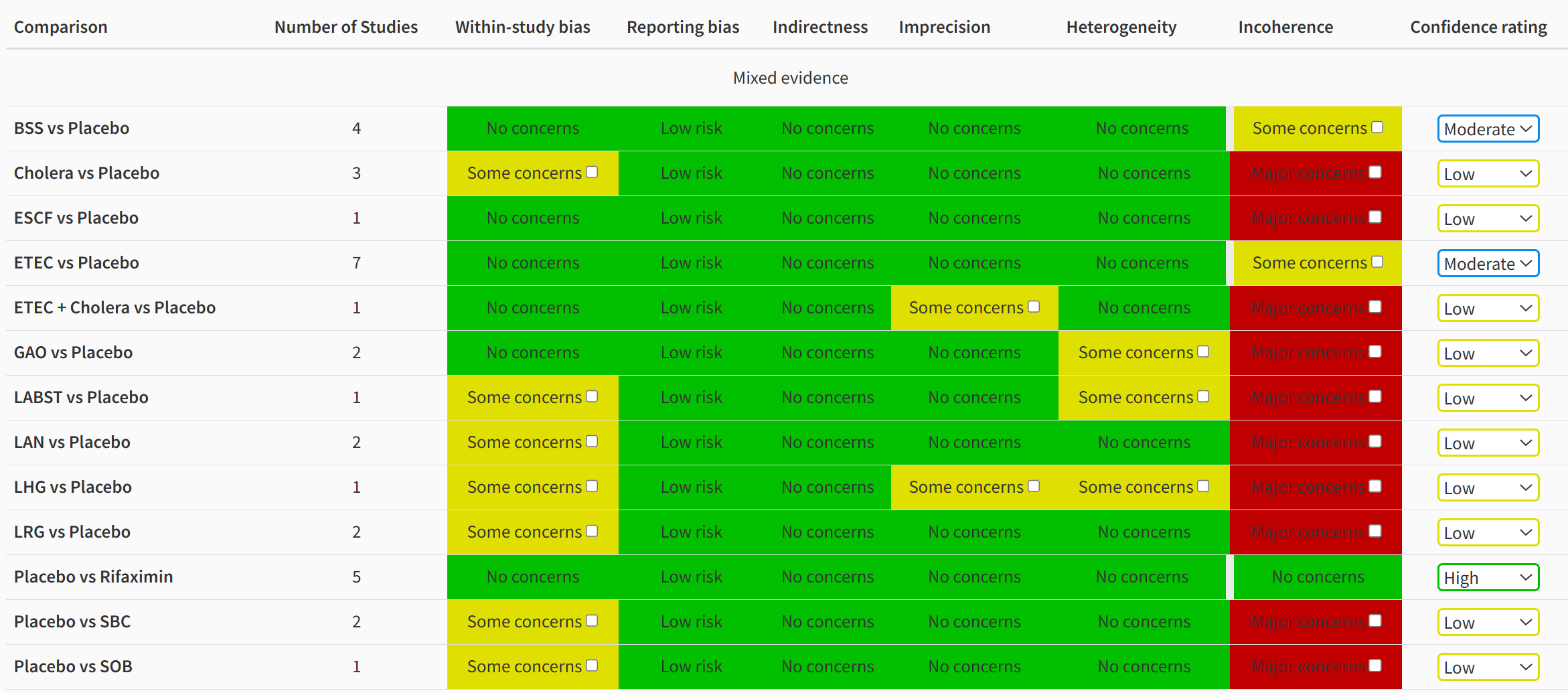
**

**
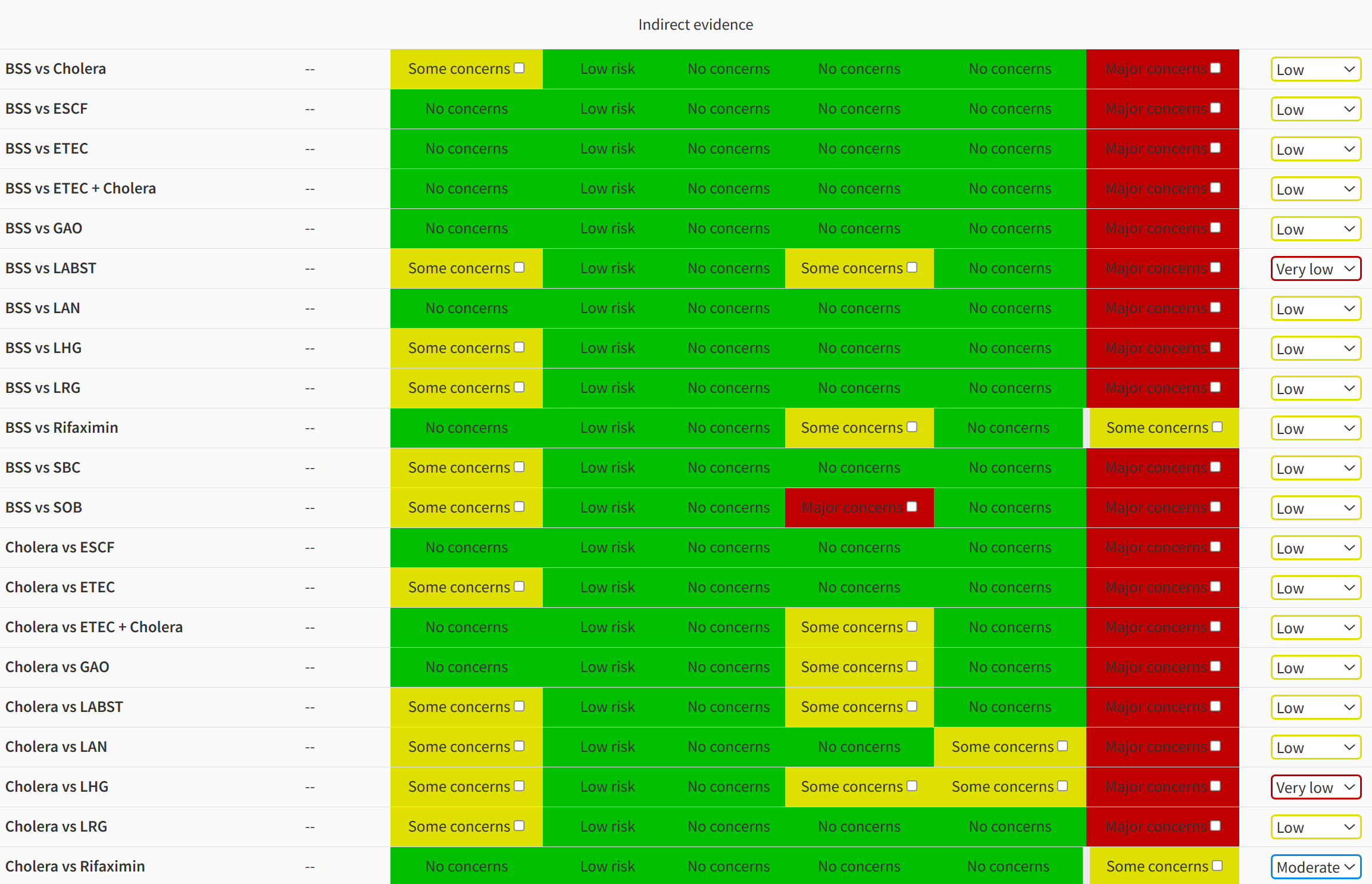
**

**
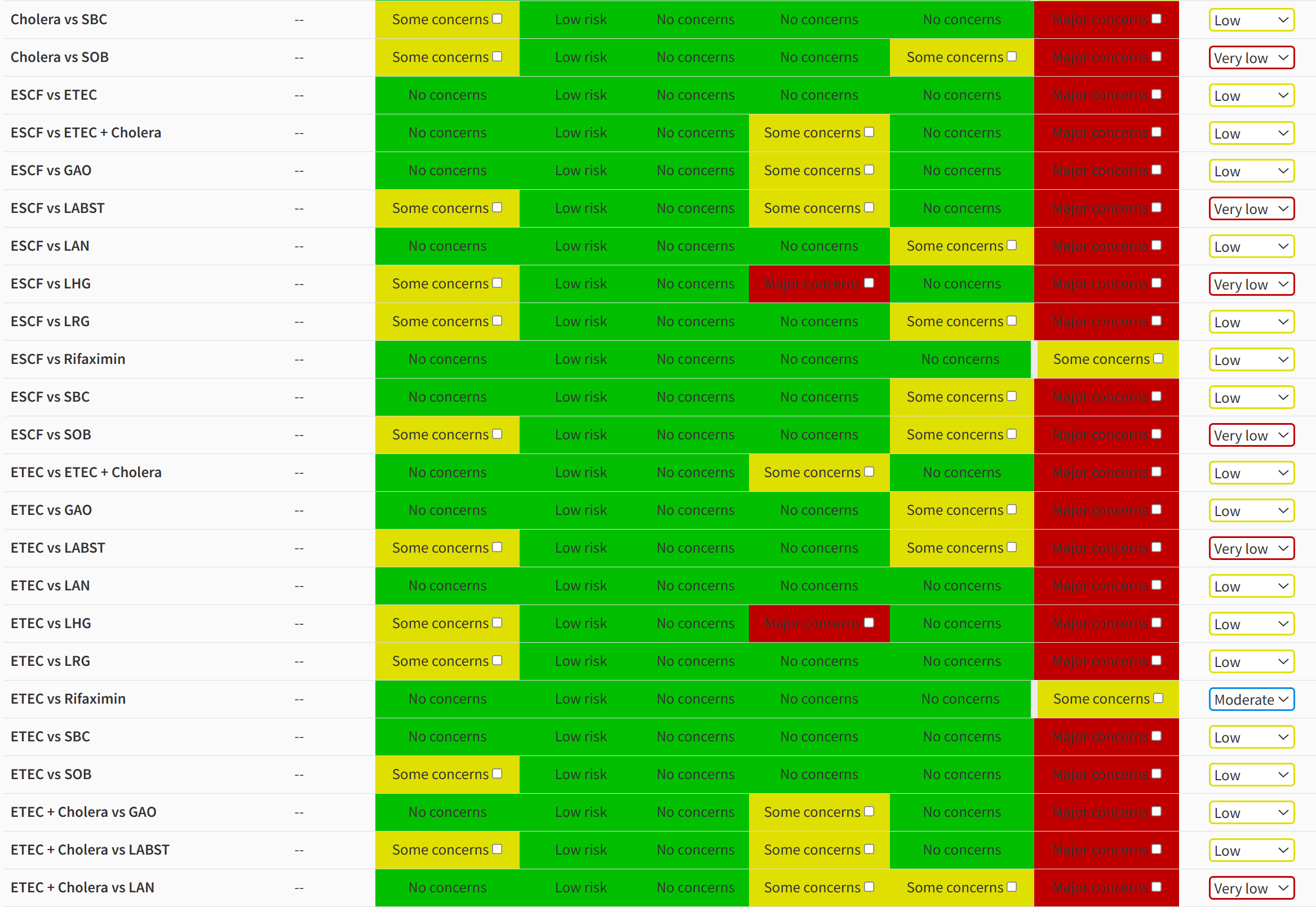
**

**
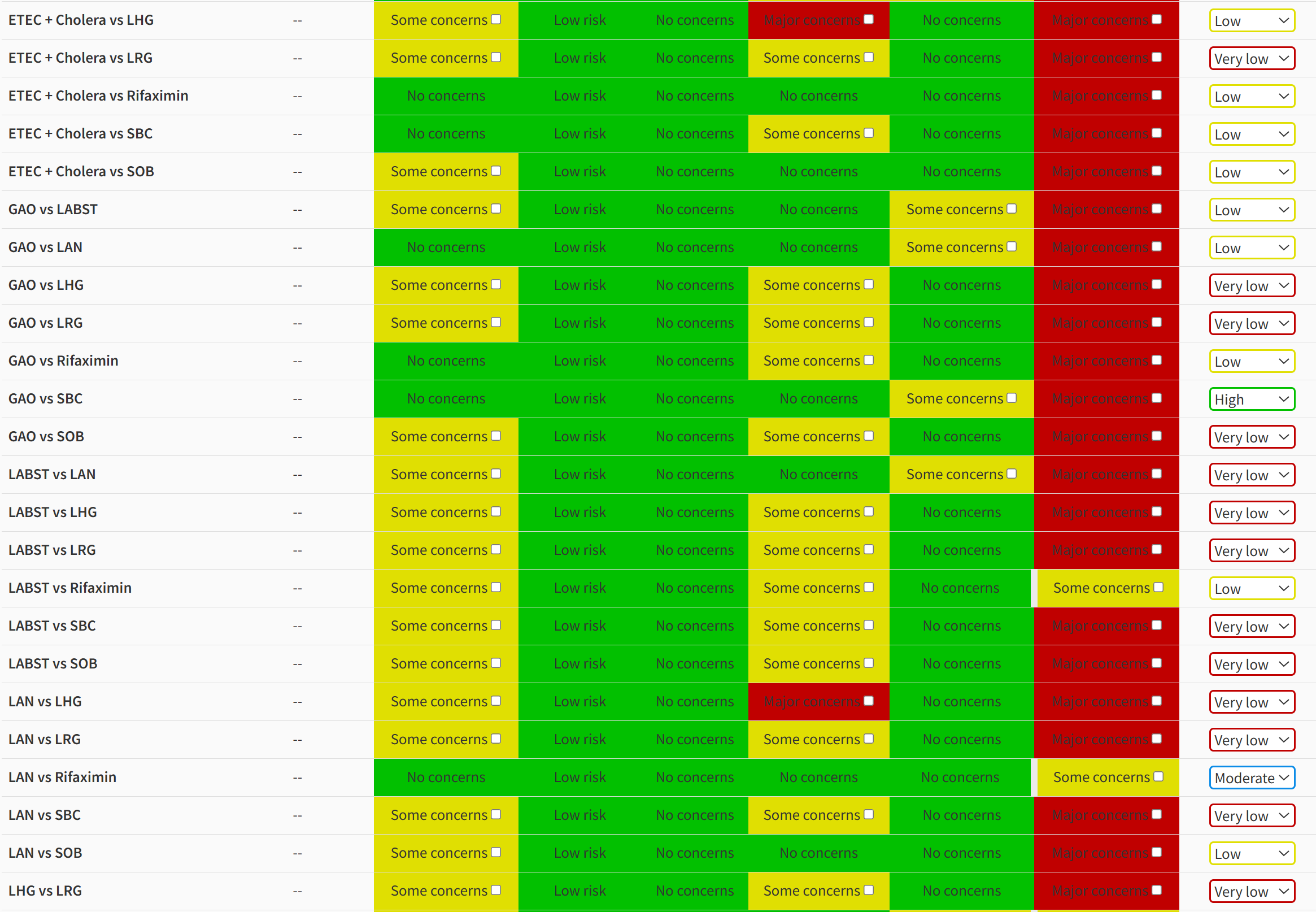
**

**
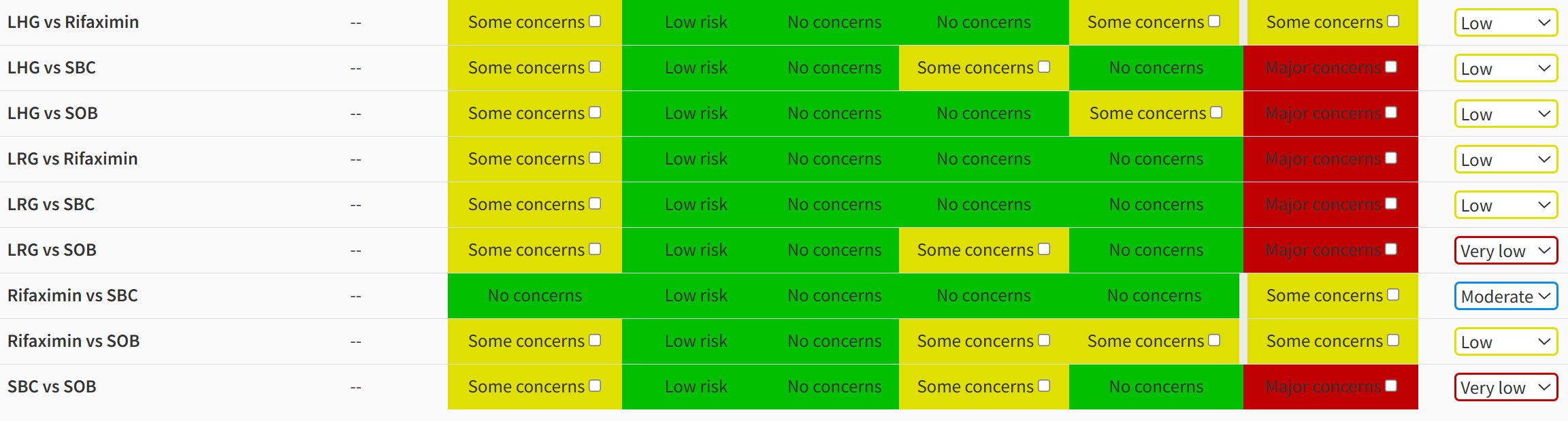
**

**Abbreviations:** BSS, Bismuth subsalicylate. ESCF, Entero. faecium SF68 + S. cerevisiae CNCM I-4444 + fructo-oliogosaccharide. ETEC, Enterotoxigenic Escherichia coli. GAO, galacto-oligosaccharide. LABST, L. acidophilus + L. bulgaricus + Biﬁdo.biﬁdum + Strept. Thermophilus. LAN, L. acidophilus nr. LHG, L. helveticus ATCC33409 + L. gasseri ATCC4962. LRG, L. rhamnosus GG. SBC, S. boulardii CNCM I-745. SOB, Sodium butyrate.

**Footnotes:** Grading of evidence was performed using Confidence in Network Meta-Analysis (CINeMA) tool.
